# Hypusinated eIF5A Promotes Ribosomal Frameshifting during Decoding of ODC Antizyme mRNA in *Saccharomyces cerevisiae*

**DOI:** 10.3390/ijms232112972

**Published:** 2022-10-26

**Authors:** Kai Halwas, Lennard-Maximilian Döring, Franziska Valentina Oehlert, R. Jürgen Dohmen

**Affiliations:** Center of Molecular Biosciences, Institute for Genetics, Department of Biology, Faculty of Natural Sciences and Mathematics, University of Cologne, 50674 Cologne, Germany

**Keywords:** polyamines, ornithine decarboxylase antizyme, hypusination, conditional mutants, DFMO, Oaz1

## Abstract

Polyamines are essential biogenic poly-cations with important roles in many cellular processes and diseases such as cancer. A rate-limiting step early in the biosynthesis of polyamines is the conversion of ornithine to putrescine by the homodimeric enzyme ornithine decarboxylase (ODC). In a conserved mechanism of posttranslational regulation, ODC antizyme (OAZ) binds to ODC monomers promoting their ubiquitin-independent degradation by the proteasome. Decoding of *OAZ* mRNA is unusual in that it involves polyamine-regulated bypassing of an internal translation termination (STOP) codon by a ribosomal frameshift (RFS) event. Using *Saccharomyces cerevisiae*, we earlier showed that high polyamine concentrations lead to increased efficiency of *OAZ1* mRNA translation by binding to nascent Oaz1 polypeptide. The binding of polyamines prevents stalling of the ribosomes on *OAZ1* mRNA caused by nascent Oaz1 polypeptide thereby promoting synthesis of full-length Oaz1. Polyamine depletion, however, also inhibits RFS during the decoding of constructs bearing the *OAZ1* shift site lacking sequences encoding the Oaz1 parts implicated in polyamine binding. Polyamine depletion is known to impair hypusine modification of translation factor eIF5A. Using a novel set of conditional mutants impaired in the function of eIF5A/Hyp2 or its hypusination, we show here that hypusinated eIF5A is required for efficient translation across the OAZ1 RFS site. These findings identify eIF5A as a part of Oaz1 regulation, and thereby of polyamine synthesis. Additional experiments with DFMO, however, show that depletion of polyamines inhibits translation across the *OAZ1* RFS site not only by reducing Hyp2 hypusination, but in addition, and even earlier, by affecting RFS more directly.

## 1. Introduction

Polyamines are essential biogenic cations derived from amino acids. The most prevalent polyamines are spermine and spermidine [1,2]. It was shown that polyamines are essential for the viability of the yeast *Saccharomyces cerevisiae*, the model organism used in the present work [3]. Polyamines have roles in multiple important cellular processes like apoptosis, replication, translation, and aging [4,5,6,7]. Regarding aging, especially the regulation of autophagy was described, where it was shown that polyamine levels, as well as the autophagy function, decrease with age. An exogenous supply of the polyamine spermidine could relieve this effect and lead to a longer lifespan in invertebrate model organisms and reduce oxidative damage in mice [8]. The effect on autophagy was recently explained by the translation regulation role of the spermidine-dependent translation factor eIF5A [9]. The synthesis of polyamines needs to be tightly regulated as too high concentrations of polyamines can be harmful. Elevated levels of polyamines are described in several cancer types providing promising targets for therapeutic approaches [10].

A critical and rate-limiting step of polyamine synthesis in yeast is the decarboxylation of ornithine, derived from arginine, by the ornithine decarboxylase (ODC). The resulting putrescine is further converted to spermidine by an aminopropyl transfer from decarboxylated S-adenosylmethionine catalyzed by spermidine synthase [11]. Spermidine can be further converted to spermine by another aminopropyl transfer mediated by spermine synthase [4,12]. The activity of ODC is dependent on the formation of a homodimer [13]. It is a protein with a very short half-life of less than 10 min in *S. cerevisiae.* The cellular abundance of ODC is mainly regulated by its interaction with ODC antizyme (OAZ), called Oaz1 in yeast [14,15,16]. This regulation is mediated by the formation of an ODC-OAZ heterodimer [16,17]. The formation of this dimer leads to ubiquitin-independent proteasomal degradation of ODC [18]. Importantly, the production of OAZ is controlled by polyamines, the products of the pathway it regulates [16]. This leads to a negative feedback regulation of ODC activity by polyamine abundance-dependent control of Oaz1 in yeast [14,19].

Regulation of OAZ by polyamines occurs via an unusual conserved mechanism involving a +1 ribosomal frameshift (RFS) during the translation of *OAZ* mRNA [20]. Similar to its human counterparts, the *S. cerevisiae OAZ1* gene consists of an open reading frame that is interrupted by a STOP codon. If this stop codon is skipped by a +1 RFS, translation continues and the main part of the full-length protein is synthesized [21]. Notably, this RFS event was shown to be promoted by high polyamine levels in yeast and human cells [14,20]. High levels of polyamines were shown to stimulate *OAZ1* mRNA translation by interacting with the nascent Oaz1 polypeptide, which prevents inhibition of translation by a pileup of ribosomes in a polysome [19]. This pileup, occurring at lower polyamine concentrations, is caused by distinct domains of nascent Oaz1. When sequence elements flanking the RFS site, and important for this mechanism, were deleted, RFS was no longer induced by high concentrations of polyamines [19]. It was, however, much earlier reported that RFS, during decoding of antizyme sequences lacking such regulatory elements in mammals, was reduced when cells were depleted for polyamines. This was achieved with the ODC inhibitor difluoromethylornithine (DFMO). The effect could be rescued by adding spermidine [22,23]. These observations suggested that RFS during decoding of *OAZ* mRNAs is not only influenced at high polyamine levels by the mechanisms described above, but also by another independent mechanism responding to very low levels as they occur upon polyamine depletion [19]. As described below, one translation factor known to depend on polyamines is eIF5A [24].

Translation factor eIF5A is a small ~17 kDa protein highly conserved in all eukaryotes [24]. Formerly thought to mainly act on translation initiation (hence the name eukaryotic translation initiation Factor 5A), it is now established that eIF5A plays a rather global role in translation elongation during the decoding of many but not all mRNAs [25,26], and also plays a role in START site selection and translation termination [27,28]. In *S. cerevisiae*, two genes called *ANB1* (also called *HYP1* or *TIF51B*) and *HYP2* (also called *TIF51A*), encode two very similar and biochemically exchangeable isoforms of eIF5A [29]. While *HYP2* is expressed under aerobic conditions, *ANB1* expression is limited to anaerobic conditions. Under regular aerobic growth conditions, no phenotype of *ANB1* deletion could be observed, whereas deletion of *HYP2* is lethal [30], indicating that the function of eIF5A is essential. EIF5A plays an important role especially during the synthesis of polyproline-rich regions, which tend to lead to ribosome stalling. Reportedly, eIF5A aids in releasing this stalling and thereby mediates translation elongation [31]. Furthermore, eIF5A appears to play a role in the nonsense-mediated decay pathway [32].

EIF5A is the only protein known to carry hypusine, an unusual post-translational modification [24]. Hypusination of eIF5A is a multi-step process, during which a 4-aminobutyl moiety, which derives from spermidine, is transferred to a specific lysine residue of eIF5A (K51 in yeast Hyp2 or Anb1). If this residue is mutated, and hypusination is thereby prevented, yeast cells are not viable [33]. Mediation of eIF5A hypusination is thought to be one critical aspect of the essential function of spermidine [34]. The first step of hypusination is mediated by deoxyhypusine synthase (DHPS), called Dys1 in *S. cerevisiae* [35,36], and results in the deoxyhypusination of eIF5A. The deoxyhypusine residue is further processed by hydroxylation mediated by deoxyhypusine hydroxylase (DOHH), called Lia1 in yeast [37,38]. The binding of eIF5A to the ribosome depends on its hypusination [39]. In *S. cerevisiae*, only deletion of *DYS1* leads to a lethal phenotype [40], whereas deletion of *LIA1* leads to slower growth [35]. It was shown that deoxyhypusinated Hyp2 is as efficient as hypusinated Hyp2 in relieving ribosomes stalled in polyproline-rich regions in yeast [31]. On the other hand, in *C. elegans* and *D. melanogaster*, loss of DOHH is recessively lethal [24]. Hypusination can be inhibited with the spermidine analog drug 1-guanidino-7-aminoheptane (GC7) [41,42]. The mode of action in translation regulation by hypusinated eIF5A was proposed based on a cryo-electron reconstruction of a Hyp2-80S ribosome complex. According to this, the hypusine residue of Hyp2 can bind to the end of a tRNA in the P-site of the ribosome, thereby stabilizing it, if it is for example destabilized by a polyproline stretch. This ensures proper positioning of the tRNA for peptide bond formation and prevents ribosome stalling [43]. EIF5A and its hypusination have been identified as relevant factors in cancer and its treatment, as well as in aging [44,45,46,47]. Spermidine has been considered as an anti-aging compound [48]. Drugs inhibiting synthesis of polyamines (such as DFMO) or the hypusine modification (GC7) have proven to be useful in the treatment of some cancer types [44,49,50].

The goal of the present study was to expand our knowledge of the mechanisms mediating the regulation of Oaz1 by polyamines. In the present study, we focused on the mechanisms acting when polyamines become limiting. We used reporter constructs containing the *OAZ1* RFS site to investigate how low RFS rates during the decoding of mRNAs derived from such constructs result from polyamine depletion. Conditional shutdown (SD) alleles of *HYP2* and *DYS1* were generated to show that hypusinated Hyp2 is specifically required to translate across the *OAZ1* RFS site. Additional experiments, however, revealed that low levels of polyamine are impinging on RFS not only by lowering levels of hypusinated Hyp2. Depletion of polyamines with DFMO, additionally, leads to lower RFS rates already much before affecting levels of hypusinated Hyp2, suggesting that decreasing levels of polyamines cause low RFS rates by multiple mechanisms.

## 2. Results

### 2.1. Reporter Constructs with the OAZ1 RFS Site

**Figure 1:**
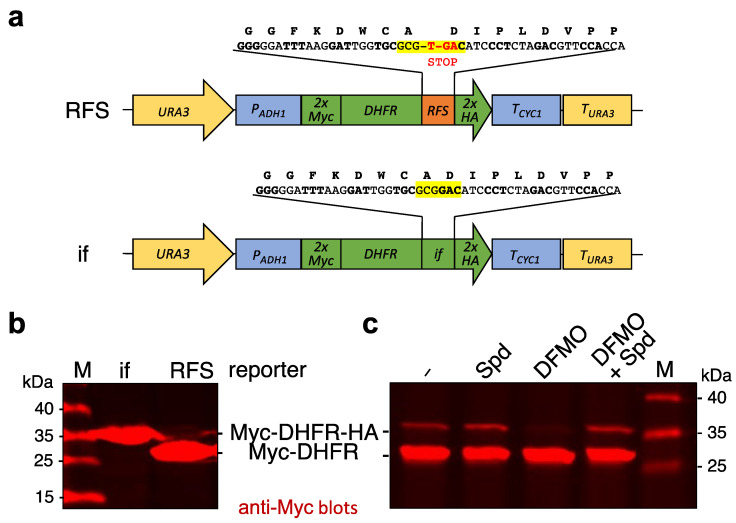
Reporter constructs with the *OAZ1* ribosomal frameshift site (RFS). (**a**) Schematic representation of reporter constructs generated. Both reporter units are integrated downstream of the endogenous *URA3* gene of *S. cerevisiae* strains. The reporter gene encompasses a sequence encoding two Myc epitopes, mouse DHFR, and either 49 nucleotides derived from the *OAZ1* gene with the RFS site at the center, or 48 nucleotides with an in-frame (if) variant of the same, followed by a sequence encoding two HA tags. Expression of these reporter genes is controlled by the *P_ADH1_* promoter and the *T_CYC1_* transcription terminator. (**b**) Detection of reporter proteins expressed either from the construct with the ribosomal frameshift site (RFS), or from its in-frame (if) variant as depicted in (**a**). The cells were grown to exponential phase in complete medium (yeast extract, peptone, dextrose/YPD; see Section 4.1). Cell extracts were analyzed by SDS-PAGE and anti-Myc Western blotting. (**c**) Western blot analysis of cells expressing the RFS reporter construct grown for 4 h in synthetic defined (SD) media with glucose and supplemented with 100 µM spermidine (Spd), 5 mM difluoromethylornithine (DFMO), or both, as indicated. Positions of the full-length reporter with 2xHA tag or of the shorter version without it, as well as of the size markers are indicated.

In order to investigate how polyamines influence ribosomal frame shifting (RFS) at the shifty site of *OAZ1* in *S. cerevisiae*, independent of their effects on translation that depend on binding to nascent Oaz1 polypeptide [19], we generated an RFS reporter construct based upon the mouse dihydrofolate reductase (DHFR) gene (Figure 1a). The reporter constructs encoded DHFR, preceded by a 2xMyc tag, and followed by 49 nucleotides of the *OAZ1* RFS site and a sequence encoding a 2xHA tag. In the middle of the RFS site, a GCG alanine codon is followed by a TGA STOP codon. Termination of translation leads to a 2xMyc-DHFR protein with a short extension and a molecular mass of 25.7 kDa. If the T nucleotide of the STOP codon at the center of the RFS site (flanked by 24 nucleotides from the *OAZ1* gene on either side) is skipped by a +1 RFS event, translation is continued with a GAC glutamate codon and extends to a STOP codon after the 2xHA sequence. The resulting full-length reporter protein has a molecular mass of 29.3 KDa. To be able to assign effects specifically to the RFS event, we generated an otherwise identical in-frame (if) control construct that lacks the above-mentioned T nucleotide and hence the premature STOP codon (Figure 1a). To avoid any effects due to gene copy number variations, the reporter constructs were stably integrated into the yeast genome downstream of the coding sequence of the *URA3* gene (Figure 1a). The two forms of the Myc-DFHR reporter protein, with and without the extension into the 2xHA sequence, can be separated by SDS-PAGE and analyzed simultaneously by anti-Myc western blotting (Figure 1b). The relative amounts of the two forms obtained with the RFS construct relates to the efficiency of RFS. As described for other reporter constructs bearing the central *OAZ1* RFS sequence but lacking other sequences encoding large parts of the Oaz1 polypeptide [19], the efficiency of frameshifting was not significantly affected by the addition of high amounts of spermidine to the media. Depletion of intracellular polyamines by the addition of the ODC inhibitor DFMO, by contrast, resulted in a clear reduction of RFS efficiency, which could be suppressed by the simultaneous addition of spermidine (Figure 1c).

### 2.2. Generation of Strains with Conditional HYP2 or DYS1 Alleles

With the RFS reporter described above in hand, we next wanted to investigate possible mechanisms explaining the reduced RFS efficiency upon depletion of polyamines. One known effect of polyamine depletion is a reduction of the hypusine modification of eIF5A (see Introduction). This translation factor is encoded by two genes in *S. cerevisiae*, namely *ANB1* and *HYP2*. *ANB1* is expressed under anaerobic conditions, whereas *HYP2* is essential at normal aerobic growth conditions (see Introduction). The encoded proteins are nearly identical with biochemically interchangeable functions. The hypusine modification on lysine residue 51 is essential for the function of both proteins and is thought to promote the interaction of eIF5A with the ribosome [30]. Using spermidine as a precursor, the generation of the hypusine modification is mediated by two yeast enzymes, Dys1 and Lia1. Only deletion of the *DYS1* gene leads to a lethal phenotype in *S. cerevisiae*, whereas deletion of *LIA1* is tolerated (see Introduction).

**Figure 2:**
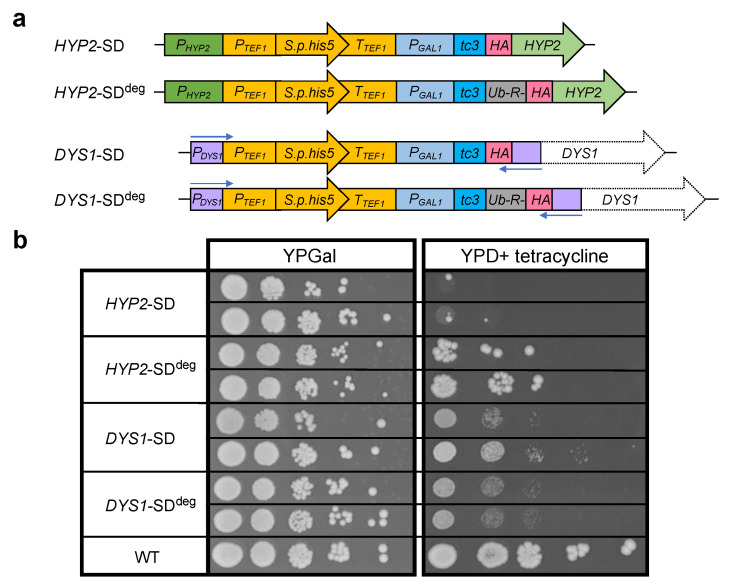
Generation and phenotypes of *HYP2* and *DYS1* shutdown (SD) strains. (**a**) Schematic representation of constructs used to generate conditional mutants. The constructs were used to replace the endogenous *HYP2* or *DYS1* loci by one-step gene transplacement in an *anb1∆* background selecting for complementation of the endogenous *his3* mutation by the *S. pombe his5* (*S.p.his5*) gene controlled by *P_TEF1_* and *T_TEF1_* (also known as *His3MX6* marker). Two constructs each were generated for *HYP2* or *DYS1.* The latter was generated by PCR using the two *HYP2* constructs as templates and long primers (shown here as blue arrows). Conditional expression enabling shutdown (SD) of the resulting genes is controlled, at the level of transcription, by the glucose-repressible *P_GAL1_* promoter and at the level of translation, by three copies of a tetracycline-controlled aptamer (tc3). All encoded Hyp2 or Dys1 proteins have an N-terminal HA tag. To render the resulting proteins metabolically unstable, one construct for each of the two proteins resulted in N-terminal fusions of degradation signals (N-degrons; Ub-R-; superscript ^deg^) leading to their degradation by the N-end rule pathway. See main text and Methods for further details. (**b**) Shown are spot assays used to evaluate the growth properties of the yeast strains created using the constructs shown in (**a**). Serial 1:10 dilutions were prepared with cells from two independent isolates for the mutants and one for the wild type, pre-grown in complete media with galactose (YPGal) and were spotted onto plates with either galactose or glucose (YPD) as a carbon source. The latter media, in addition, contained 250 µM tetracycline. Pictures were taken after 3 days of incubation at 30 °C.

In order to investigate the possible roles of eIF5A and its hypusination, we wanted to create a set of yeast strains with conditional *HYP2* and *DYS1* alleles (Figure 2a).

An often-applied strategy is the use of alleles encoding modified versions of a protein carrying conditional temperature-controlled degrons [51,52]. The strategies described in these studies employ the fusion of relatively large protein domains to the N-terminus of a protein thereby promoting degradation of the resulting fusion protein at a higher temperature. These approaches, however, did not prove to be applicable to Hyp2 because the resulting fusion proteins did not sustain viability. We, therefore, decided to combine several previously described alternative approaches to achieve a stringent shutdown (SD) of *HYP2* expression. To achieve rapid transcriptional repression of *HYP2*, we placed it under the control of the galactose-induced and glucose-repressed promoter of the *S. cerevisiae* galactokinase gene (*P_GAL1_*) [53]. In addition, to be able to also stop translation of pre-existing mRNAs, we inserted an aptamer sequence reported to form three tetracycline-stabilized hairpin structures (tc3) at the level of the mRNA [54] between *P_GAL1_* and the translation START codon. In the presence of tetracycline, these RNA aptamers inhibit translational initiation. This two-pronged approach was designed to simultaneously and effectively terminate transcription and translation by the addition of glucose and tetracycline, respectively, to the culture media. In an attempt to moreover deal with a possible long half-life of Hyp2, we additionally added a very short N-terminal degradation signal (N-degron) to the protein. This was achieved by fusing the *HYP2* coding sequence in frame downstream to a sequence encoding ubiquitin (Ub) followed by an arginine (R) residue. Ub is rapidly cleaved upon synthesis of the fusion protein in the cell by deubiquitylating enzymes, thereby exposing R at the N-terminus of the resulting protein, which serves as a constitutive degradation signal within the N-end rule pathway [55]. Both constructs carried a short sequence encoding an HA epitope at the N-terminus of the resulting proteins. The constructs were, in addition, linked to a selectable marker (*His3MX6*) [56] (Figure 2a, upper part), and used to replace the endogenous copies of *HYP2* in an *anb1∆* background. The latter was chosen to exclude a possible leakiness of the phenotype caused by an unwanted expression of *ANB1.*

The established SD constructs for *HYP2* described above bearing *His3MX6*-*P_GAL1_*-*tc3-HA* or *His3MX6*-*P_GAL1_*-*tc3*-*Ub-R-HA* modules were then used as a starting point in a PCR-based approach to create similar constructs for *DYS1*. Using long oligonucleotides attaching ~150 Bp homology to the 3′ end of *P_DYS1_* at one end, and to the 5′ end of the *DYS1* ORF at the other end, such *DYS1*-specific SD constructs were amplified, initially cloned in plasmids, and then used for replacing the endogenous *DYS1* locus of *S. cerevisiae* strains (Figure 2a, lower part).

Using the four distinct constructs depicted in Figure 2a, four types of strains were generated, which were initially selected on galactose media lacking histidine. The growth properties of the resulting strains were compared to those of the congenic wild type on complete media with galactose or with glucose and tetracycline. On the galactose plate, the different SD strains, with one exception, showed growth behavior similar to the wild type. The exception was the *HYP2*-SD^deg^ strain, for which both isolates tested displayed a smaller colony size indicative of a slower growth rate. This observation indicated that the short-lived variant of Hyp2 bearing the N-degron was insufficient to sustain wild-type growth on this medium. Again, with the exception of the same isolates, all other SD strains displayed very strong growth impairment on the glucose + tetracycline plate. The relatively small number of large colonies observed for the *HYP2*-SD^deg^ strains was likely due to spontaneous mutations that either interfered with the transcriptional or translational repression mechanisms or with protein degradation by the N-end rule pathway [57]. The observation that such mutants were observed more frequently than for the other strains can be explained by their positive selection already in galactose media due to the growth impairment caused by the *HYP2*-SD^deg^ allele (see above). We, therefore, chose the *HYP2*-SD mutants, which displayed a rather tight shutdown phenotype, for further experiments. For *DYS1*, by contrast, we picked the *DYS1*-SD^deg^ mutants for further studies because their shutdown phenotype was slightly more stringent than that of *DYS1*-SD mutants (Figure 2b). Note that the SD phenotype of *HYP2*-SD mutants appears tighter than that of *DYS1*-SD^deg^ mutants, despite the added degron sequence in the latter allele. This can in part be explained by the fact that a loss of Dys1 function more indirectly affects viability by eventually depleting hypusinated Hyp2. In addition, it appears that low levels of Dys1 acting catalytically on Hyp2 are sufficient to sustain cell viability for quite some time.

### 2.3. Control and Detection of Hypusinated Hyp2

**Figure 3:**
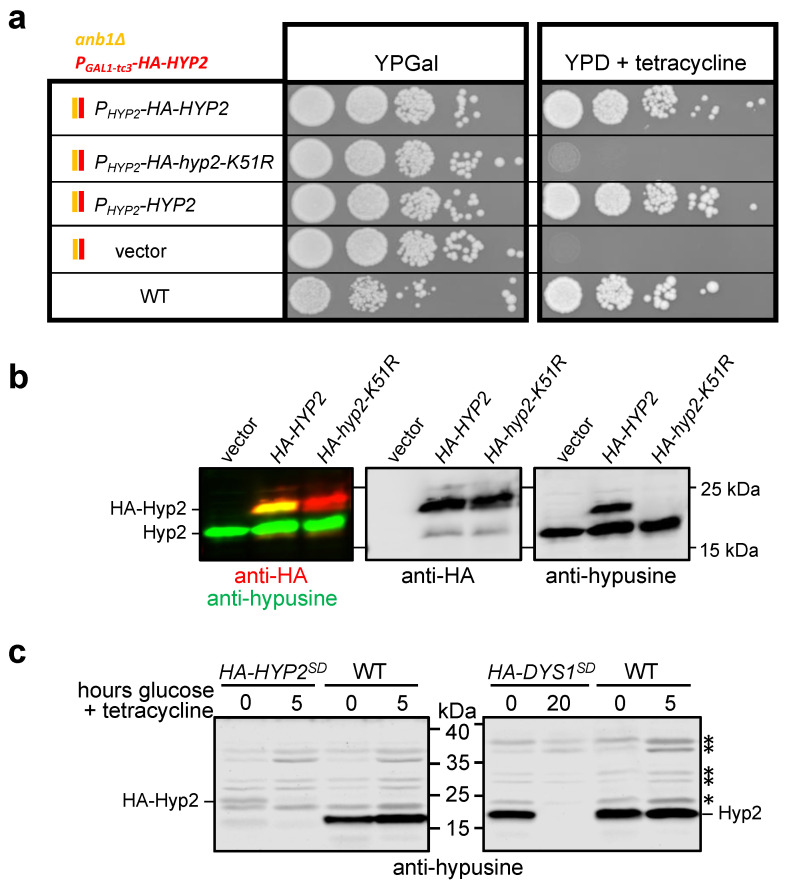
Analysis of hypusinated Hyp2. (**a**) Shown is a spot assay of serial dilutions (1:10) of an *anb1∆ HYP2*-SD strain transformed either with an empty *CEN/LEU2* vector, or the same vector expressing untagged *HYP2*, *HA-HYP2*, or *HA-hyp2-K51R*. The latter encodes a mutant version of Hyp2 lacking lysine 51, the site of hypusination. Transformants were selected on galactose media lacking leucine and then spotted on the indicated media. Pictures were taken after 3 days of incubation at 30 °C. (**b**) Simultaneous anti-HA (red) and anti-hypusine (green) western blot analysis of extracts from a wild-type strain transformed either with an empty vector or the same vector encoding *HA-HYP2* or *HA-hyp2-K51R* (left panel). The middle and right panels show the individual signals in grayscale. (**c**) Shown is an anti-hypusine western blot analysis to monitor the disappearance of hypusinated Hyp2 after shutting down expression of either *HYP2* (*HYP2*-SD) for 5 h, or *DYS1* (*DYS1*-SD^deg^) for 20 h, by growing the cells in YPD with 250 µM tetracycline. A congenic wild-type strain was treated the same way for 5 h. *, non-specific bands.

The established *HYP2*-SD *anb1∆* strain enabled the testing of the functionality of *HYP2* introduced on centromeric (*CEN*) plasmids. We took advantage of this in order to first test whether the N-terminal HA tag would have any detectable effect on cell growth. Cells transformed with plasmids expressing untagged *HYP2* from its authentic promoter were indistinguishable in their growth properties from one expressing an HA-tagged variant from an otherwise identical plasmid (Figure 3a). This observation indicated that in contrast to larger domains (see above), the smaller HA tag did not interfere with the Hyp2 function. We also confirmed that lysine residue 51 (K51), known to be the site of hypusination [30], is essential for Hyp2 function. A plasmid encoding HA-Hyp2-K51R was unable to sustain the growth of the *HYP2*-SD strain on media with glucose and tetracycline (Figure 3a).

Using the same plasmids introduced into a wild-type strain, we tested the specificity of an antibody against hypusinated eIF5A. As shown in Figure 3b, this antibody specifically recognized endogenous hypusinated Hyp2 as well as plasmid-encoded HA-Hyp2, but not HA-Hyp2-K51R. We also tested this antibody on the *HYP2*-SD and *DYS1*-SD^deg^ strains (Figure 3c). The signals for hypusinated HA-Hyp2 and endogenous untagged Hyp2 were detectable in these strains when grown in galactose media, but were nearly undetectable when cells were grown in media with glucose and tetracycline. We conclude that the antibody used allowed very specific detection of hypusinated Hyp2 in yeast cells and that the the *HYP2*-SD or the *DYS1*-SD^deg^ strains can be used to deplete Hyp2 or its hypusinated form, respectively.

### 2.4. Hypusinated Hyp2 Promotes Ribosomal Frameshifting during Decoding of the OAZ1 RFS Site

**Figure 4:**
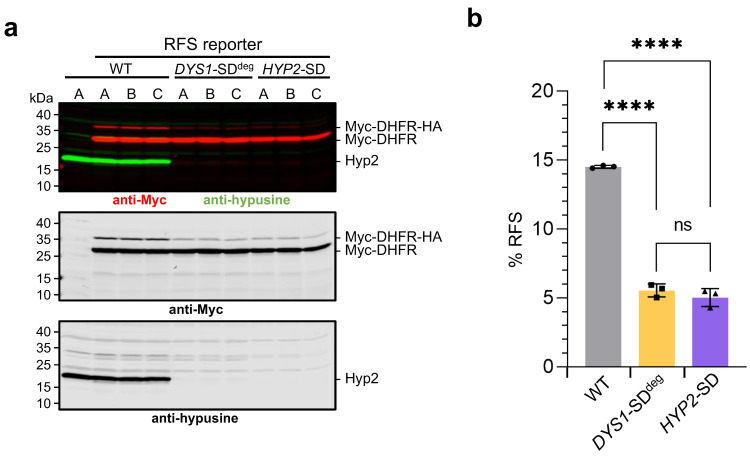
*DYS1* and *HYP2* shutdown leads to an inhibition of *OAZ1* ribosomal frameshifting. (**a**) Western blot analysis of RFS reporter levels in the indicated shutdown (SD) strains compared to the wild type (WT). The WT and the *P_GAL1_-tc3-HA-HYP2* (*HYP2*-SD) strains were incubated for 8 h in YPD with 250 µM tetracycline. The *P_GAL1_-tc3-UB-R-HA-DYS1* (*DYS1*-SD^deg^) strains were incubated for 20 h in the same media with sequential dilutions to prevent entry into stationary phase due to nutrient deprivation. The experiment was performed with three independent isogenic spore clones (A, B, C) for each genotype. To confirm the specificity of the reporter detection by the Myc antibody, a WT strain without RFS reporter was analyzed as a control (first lane). Extract proteins were analyzed by SDS-PAGE and western blotting with simultaneous detection of Myc (red) and hypusine signals (green) (top panel). The middle and bottom panels show the individual signals in grayscale. (**b**) Shown is a quantitative analysis of ratios of the signals for the full-length reporter resulting from ribosomal frameshifting to the signals for the shorter reporter resulting from premature translation termination at the internal STOP codon. The relative amounts of the full-length reporter (in % of the total Myc-DHFR signal) are depicted as % RFS. Individual values are shown as dots, squares, or triangles. The bars represent the means of values obtained from the three independent spore clones analyzed for each genotype in (**a**). Standard deviations (SD) are indicated by error bars. Four asterisks indicate significant differences with *p*-values < 0.0001; ns, not significant. Statistical significance was determined with unpaired and two-sided Student’s *t*-tests. Numbers obtained from signal quantification and calculation of SD and significance are shown in Table S1. The full-size blot and total protein stain loading control are shown in Supplementary Figure S1.

The established SD alleles *HYP2*-SD or *DYS1*-SD^deg^ were combined with the stably integrated reporter constructs described in Section 2.1 by mating and tetrad dissection. Multiple congenic spore clones were isolated for each genotype and used to perform a western blot analysis to test whether hypusinated Hyp2 has a role in ribosomal frameshifting (RFS) at the shifty site taken from the *OAZ1* gene. Wild-type cells and the different SD mutants were pre-grown in galactose media and then shifted to glucose + tetracycline media either for 8 h (wild type and *HYP2*-SD) or for 20 h (*DYS1*-SD^deg^). Preliminary experiments had shown, consistent with the different growth behavior observed on plates (see above, Figure 2b), that the *DYS1*-SD^deg^ allele needed a significantly longer SD time to be as effective as *HYP2*-SD. Reporter levels were then analyzed in extracts by quantitative western blot analysis (Figure 4). Similar to what was introduced above (Figure 1), premature termination without RFS during translation of the reporter mRNA at an internal STOP codon led to a shorter form of the reporter, whereas RFS enabled the continuation of the synthesis, adding a segment of ~3.6 kDa including a 2xHA tag. The relative amounts of these two forms can be used to determine the RFS rate, expressed as % of the full-length relative to the sum of both forms. In the wild type, the RFS rate was ~14.5%. By contrast, RFS rates were ~5%, both in the *HYP2*-SD mutants after 8 h in YPD + tetracycline and in the *DYS1*-SD^deg^ strains after 20 h in the same media. Importantly, the overall efficiency of reporter mRNA translation was unaffected (Figure 4a and Figure S1a) by the application of SD conditions. Moreover, when we performed the same treatment with otherwise identical strains expressing the in-frame variant of the reporter, no similar reduction of the full-length reporter protein compared to the wild type was to be observed after the application of SD conditions (Figure S1b). Together, these results demonstrated that depletion of Hyp2, or inhibition of Hyp2 hypusination, both cause a specific inhibition of RFS during decoding of the reporter mRNA at the *OAZ1* shifty site, without any general effects on the translation of the reporter mRNA. We conclude, hypusinated Hyp2/eIF5A is required for efficient RFS during decoding of *OAZ*1 mRNA.

### 2.5. Polyamines Affect RFS Not only by Promoting Hyp2 Hypusination

**Figure 5:**
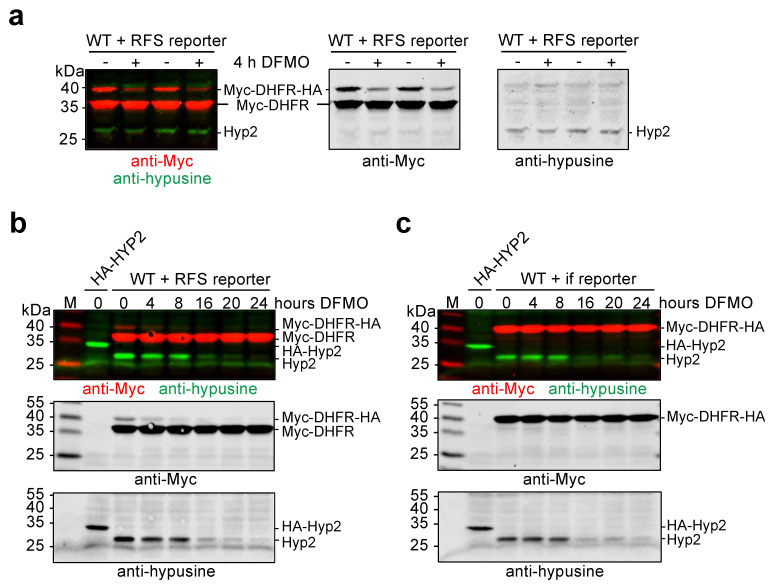
Polyamine depletion inhibits ribosomal frameshifting earlier than affecting Hyp2 hypusination (**a**) Western blot analysis of RFS reporter and hypusinated Hyp2 levels. Two independent spore clones of wild-type (WT) cells expressing the RFS reporter were grown in synthetic SD media and incubated either with or without 5 mM DFMO for four hours. (**b**) Same as in (**a**), except that DFMO was added at time point 0, and samples were taken after the indicated times for up to 24 h later. (**c**) Same as in (**b**), except that the cells expressed the in-frame (if) reporter. Extract proteins were analyzed by SDS-PAGE and western blotting with simultaneous detection of Myc (red) and hypusine signals (green). Grayscale panels separately show the signals detected with the individual antibodies. Positions of the relevant polypeptides and the size markers (M) are indicated.

Having established that Hyp2 hypusination is of critical importance for RFS during decoding of the *OAZ1* RFS site, we next asked the question of whether inhibition of RFS by DFMO (Figure 1c) correlated with depletion of hypusinated Hyp2. To address this question, we first employed prototrophic wild-type cells carrying the RFS reporter construct. Cells from two independent spore clones were incubated in synthetic media for 4 h, either in the absence or presence of 5 mM DFMO. Western blot analysis of reporter polypeptide levels and of hypusinated Hyp2 showed a strong reduction of the full-length reporter polypeptide in the extracts from cells treated with DFMO, indicating that RFS efficiency was strongly reduced (Figure 5a). At the same time, no apparent reduction of hypusinated Hyp2 was to be observed.

In order to investigate at which point DFMO treatment would result in the reduction of hypusinated Hyp2 and how this affects RFS, the experiment was repeated with strains either expressing the RFS reporter or the in-frame reporter. These cells were cultivated for up to 24 h in the presence of 5 mM DFMO. The cells were kept growing by diluting them with the same synthetic media at each assay time point. Samples were taken at various time points and analyzed for the levels of the different forms of the reporter protein and of hypusinated Hyp2. In these experiments, DFMO treatment, again, inhibited RFS already after 4 h much before a strong reduction of hypusinated Hyp2 was to be observed (Figure 5b). At later time points (16 to 24 h) of DFMO treatment, levels of the full-length reporter resulting from the continued translation after RFS further decreased coinciding with the depletion of hypusinated Hyp2. These findings indicated that depletion of polyamines affects translation across the *OAZ1* RFS site even before Hyp2 function is impaired, but that upon further depletion of polyamines, likely the reduction of hypusinated Hyp2 leads to an even stronger inhibition of RFS. In conclusion, the feedback regulation of polyamines synthesis via regulation of *OAZ1* mRNA translation occurs at multiple levels, one of which is the control of Hyp2 hypusination.

## 3. Discussion

### 3.1. Reporter Constructs to Study Effects of Polyamine Depletion on Decoding of OAZ1 RFS Site

The goal of the present study was to investigate how the depletion of polyamines impinges on the decoding of the *OAZ1* RFS site. To investigate this, we generated a couple of reporter constructs, which we introduced in single copy stably into the genome of *S. cerevisiae* strains by placing them downstream of the coding sequence of a selectable marker gene (*URA3*). The reporter genes contained only 48 or 49 nucleotides of the sequence around the *OAZ1* RFS site, respectively, for the in-frame control construct or the RFS construct. The RFS site was only followed by a short stretch encoding a 33 amino acid residue sequence including a 2xHA tag. As a result, the shorter polypeptide resulting from premature termination at the STOP codon in the RFS site, and the full-length reporter protein produced if RFS leads to skipping of the STOP codon and continuation of translation, run in the same area of the gel and can easily be analyzed together by quantitative western blotting (Figure 1). Another important reason for the design of the reporters was that we wanted to exclude parts of the Oaz1 protein that were shown to be important to sense high concentrations of polyamines co-translationally to stimulate *OAZ1* mRNA translation in polysomes [19]. As a consequence, these reporter constructs do not respond to the application of high levels of polyamines in a similar way as a full-length *OAZ1* gene does (Figure 1c) [19]. They do, however, respond to a depletion of polyamines by application of the ODC inhibitor DFMO (Figure 1c and Figure 5).

### 3.2. Design of HYP2 and DYS1 Shutdown Alleles

Depletion of polyamines is known to cause a reduction in the levels of hypusinated translation factor eIF5A/Hyp2. Therefore, we asked whether depletion of Hyp2 or its hypusinated form is underlying the observed reduction of RFS during reporter mRNA decoding. We developed new combinatorial strategies to conditionally control the levels of Hyp2 or of Dys1/deoxyhypusine synthase. Because the addition of bulky domains driving conditional proteolytic targeting of the encoded proteins appeared inappropriate, we went for conditionally controlling expression both at the level of transcription, by using the glucose-repressible *P_GAL1_* promoter, and of translation, by introducing tetracycline-controllable aptamer sequences (tc3) [54]. These alleles were used to replace the endogenous gene copies of *HYP2* and *DYS1* yielding strains that enabled a rather efficient shutdown (SD) of expression by the addition of glucose and tetracycline. For depletion of the Dys1 protein, it turned out to improve the stringency of the SD condition further when a very short constitutive degron was appended to its N-terminus. The same strategy, by contrast, was not helpful for Hyp2, because the resulting strains were already impaired in their growth at the permissive condition, which resulted in the selection of spontaneous mutants that had overcome the SD phenotype. These results indicate that such combinatorial strategies to achieve conditional phenotypes need to be independently tested for each gene to be studied. As the examples of this study show, the kinetics of establishing an SD phenotype can be very different for distinct genes, which depends on multiple parameters such as the stability of the encoded protein, the levels of it required for maintenance of cellular function, and whether the protein acts directly or indirectly in a process. The *HYP2-SD* phenotype was more stringent and more rapidly effective than that of *DYS1*-SD^deg^, although the former lacked a sequence for an N-degron. SD times of 8 h were sufficient to effectively deplete hypusinated Hyp2, whereas much longer SD times (we used 20 h) were required for *DYS1*-SD^deg^. A likely explanation is that very low levels of Dys1 protein are sufficient to sustain critical levels of Hyp2 hypusination. The established SD alleles of genes can also be employed to screen for temperature-sensitive (ts) alleles of a gene of interest or to introduce known such alleles into a genetic background of choice. Transformants in which such ts alleles have replaced the *His3MX6*-marked SD allele can readily be selected on glucose + tetracyline media, followed by verification of the ts phenotype and loss of the *His3MX6* marker.

### 3.3. Hypusinated Hyp2 Is Required for Efficient Translation across the OAZ1 RFS Site

We employed congenic sets of *HYP2*-SD and *DYS1-*SD^deg^ strains expressing either the RFS or the in-frame reporters to investigate whether hypusinated Hyp2 is important for the translation of either of the reporters. The results clearly show that Hyp2 and its hypusination are required for efficient synthesis of the full-length version of the reporter protein derived from the construct with the *OAZ1* RFS site (Figure 4). Synthesis of the overall amounts of reporter protein either from the RFS or the in-frame reporter constructs, by contrast, was not affected in a similar way by SD of *HYP2* or *DYS1* (Figure S1) indicating that hypusinated Hyp2 was specifically required for translation across the *OAZ1* RFS site of the reporter mRNA, but not in general for its translation. These data are in line with an earlier observation that eIF5A affects frameshifting during translation across a shifty site derived from the yeast Ty1 retrotransposon [25]. Our findings establish that hypusinated eIF5A can act as one component in regulatory feedback regulation of polyamine synthesis. When polyamines are sufficiently low, depletion of hypusinated Hyp2 will cause a reduction in Oaz1 production and hence stabilization of ODC (see Introduction). Given the high conservation of eIF5A roles in translation [24], and of the polyamine regulation of ribosomal frameshifting during *OAZ* mRNA decoding [15,16], it seems likely that a critical role of eIF5A in OAZ synthesis, as described here for *S. cerevisiae*, is also relevant in humans. Regulation of OAZ levels via eIF5A entails the possibility of additional regulatory inputs aside from polyamine depletion. Alterations of eIF5A levels or its modification upon other changes in cellular physiology, for example during development or the cell cycle, or upon stress, could mediate alterations by adjusting translation of selected mRNAs including *OAZ*, thereby adjusting ODC and ultimately polyamine levels [58].

### 3.4. Polyamines Are Required to Promote Translation across the OAZ1 RFS Site

The results discussed above were in line with the possibility that the main effect of polyamine depletion on translation through the *OAZ1* shifty site is via reducing levels of hypusinated Hyp2. Additional experiments with a four-hour treatment of cells with ODC inhibitor DFMO revealed that synthesis of the full-length reporter protein from the RFS construct was already strongly reduced before levels of hypusinated Hyp2 dropped (Figure 5a). This result indicated that polyamines have an additional, probably more direct role in promoting translation across the STOP codon in the *OAZ1* RFS site. How exactly polyamines impinge on the RFS event is not fully understood and may involve interactions with the ribosome, tRNAs, the transcript, or termination factors [6,16,21,59].

In order to test whether the reduction of hypusinated Hyp2 upon longer depletion of polyamines has an additional effect on RFS, we performed additional tests with rather long DFMO treatment (up to 24 h). These experiments revealed that somewhere between 8 and 16 h of treatment, the levels of hypusinated Hyp2 eventually dropped, which coincided with a further reduction of the full-length RFS reporter protein.

Together, our results indicate that low levels of polyamines are sensed at least at two levels involved in the control of translation across the *OAZ1* RFS site. These are part of an even more complex overall regulation of Oaz1 levels by polyamines (see below).

### 3.5. Polyamines Act at Multiple Levels in a Feedback Control of Their Own Synthesis

Taking the results reported here together with those from previous reports, a complex picture of how polyamines mediate a feedback control of their own synthesis via regulation of Oaz1 and ODC levels emerges. When levels of polyamines such as spermidine are high, they bind to nascent Oaz1 polypeptide to prevent the pileup of ribosomes on *OAZ1* mRNA thereby increasing the efficiency of synthesis and RFS [19]. In addition, the binding of spermidine to Oaz1 increases its efficiency in targeting ODC for ubiquitin-independent degradation [19,60]. When polyamine levels become limiting, two mechanisms dissected in the present study operate to limit the synthesis of Oaz1. First, a reduction of RFS is observed, which apparently occurs much before a clear reduction of levels of hypusinated Hyp2 is to be observed (Figure 5a,b). This reduction, furthermore, is independent of parts of the Oaz1 polypeptide required to sense polyamines [19] as they are missing in the reporter constructs used. These findings suggest that limiting polyamine levels are directly influencing RFS possibly by altering either recognition of the alanine codon by a tRNA with a near-cognate anti-codon, or by affecting STOP codon recognition. When polyamine levels drop further, this eventually leads to a depletion of hypusinated Hyp2, which is required for efficient translation across the *OAZ1* RFS site (Figure 4), and as a result to a further reduction of RFS (Figure 5b). In a consideration of kinetics, a fast consequence of polyamine limitation is a direct and negative one on RFS. If polyamine depletion holds on for a longer period, this will lead to a depletion of hypusinated Hyp2, further reducing RFS to very low levels such that hardly any Oaz1 is produced. This, in turn, results in a stabilization of ODC and an increase in polyamine synthesis. Oaz1 is not the only target of such a feedback regulation in the polyamine metabolism. Regulation of the polyamine transporter Hol1, for example, is under translational auto-regulation involving upstream ORFs and a polyamine- and eIF5A-dependent control of its STOP codon recognition [61].

Knowledge of the details of these complex regulatory mechanisms can help to understand why combinatorial therapies, which employ DFMO to inhibit ODC, and GC7 to inhibit eIF5A hypusination, are particularly effective in the treatments of certain cancers [62].

## 4. Materials and Methods

### 4.1. Yeast Methods

Yeast peptone (YP) and synthetic defined (SD) media were prepared, either containing 2% dextrose (=glucose) or 2% galactose, and with or without 250 µM tetracycline. *S. cerevisiae* strains used are all prototrophic derivatives of WCG4a [63] (listed in Table S2). To obtain prototrophic derivatives of this strain, the *leu2–3,112* mutation was reverted by transformation with a fragment containing the wild-type *LEU2* gene, and the *his3–11,15* mutation of strains not carrying a *His3MX6-*marked conditional *HYP2* or *DYS1* allele was reverted by transformation with a fragment containing the wild-type *HIS3* gene. Multiple independent congenic spore clones were selected for different combinations of alleles and integrated reporter genes after crossing and tetrad analyses. The growth of yeast cells in liquid media was monitored by photometric measurement of optical density at 600 nm (OD_600_). Plasmids used for yeast transformation are listed in Table S3. Centromeric plasmids carrying different variants of the *HYP2* gene were derivatives of pRS315 [64].

Haploid yeast cells were prepared by tetrad dissection after the transformation of diploid cells or crossing. Sporulation was induced by incubation of diploid cells in sporulation media (0.005% zinc sulfate, 1% potassium acetate) for 5–7 days. The abundance of tetrads was monitored by microscopy. The culture containing tetrads was centrifuged and resuspended in 0.5 mg/mL zymolyase and incubated at room temperature for 10 min. The tetrads were then dissected on YP plates with galactose and analyzed further for the presence of desired markers.

To compare growth properties, yeast cells were spotted onto agar plates containing different media. For that, cultures were incubated to exponential growth. Droplets of undiluted culture (OD_600nm_~0.4) and of four serial 1:10 dilutions were used for each strain tested. Images were taken after 3 days of growth at 30 °C.

### 4.2. Generation of Ribosomal Frameshift (RFS) Reporters

To generate strains with stable genomic integration of reporter constructs, plasmids were first generated in the background of a derivative of pUC21 [65] (pLD35), in which the *URA3* ORF followed by a polylinker sequence and the terminator region of the *URA3* gene (T*_URA3_*) were inserted. This polylinker was used to insert a *P_ADH1_*-2xMYC-*DHFR*-RFS*^OAZ1^*-2xHA-*T_CYC1_* unit that encompasses the promoter of the *ADH1* gene controlling expression of a 2xMyc tag linked 5′ to the mouse dihydrofolate reductase (DHFR) gene fused in frame to 49 Bp of the yeast *OAZ1* gene encompassing the ribosomal frameshift (RFS) site followed by a sequence encoding a 2xHA epitope tag, resulting in plasmid pFO1. pFO2 was identical to this plasmid, except that an in-frame (if) variant resulted from a single T nucleotide deletion in the RFS site. The resulting reporter constructs are schematically depicted in Figure 1a. Sequences of the plasmids are shown in Figure S2. The inserts of pFO1 or pFO2 were released from the pUC21 backbone by restriction with NotI, and the cleaved DNA was used to transform *ura3-52* yeast cells selecting for uracil prototrophic transformants. Correct stable insertion of the expression cassette in the yeast genome downstream of the *URA3* ORF was verified by PCR.

### 4.3. Generation of HYP2 Shutdown (SD) Strains

To generate yeast strains in which levels of the Hyp2 protein could be controlled by multiple means, first, two different plasmids (pKH15 and pKH16) were generated in the background of pUC19 [66] using DNA segments produced by PCR. Both plasmids encompassed an upstream segment from the 5′ untranslated region of the *HYP2* gene, an expression unit (known as *His3MX6*) encoding the S*. pombe his5^+^* gene (complements the *his3∆-200* mutant of the *S. cerevisiae* strains used) taken from plasmid pFA6a-His3MX6 [56], the promoter of the galactose-inducible and glucose-repressible *GAL1* gene (*P_GAL1_*), a sequence encompassing three tetracycline-stabilized hairpin loops (tc3) formed at the mRNA level [54], and the *HYP2* ORF with some downstream sequence (*T_HYP2_*). In pKH15, the *HYP2* ORF was fused in frame downstream of a sequence encoding an N-degron (Ub-R) derived from pPW66R [51] and an HA tag. Because the N-terminal ubiquitin (Ub) is cleaved rapidly by endogenous deubiquitylating enzymes, an arginine (R) residue is exposed at the N-terminus of the resulting protein leading to its rapid degradation by the ubiquitin-dependent N-end rule pathway [55]. By contrast, in the otherwise identical plasmid pKH16, the Ub-R sequence is missing, such that a more stable version of the HA-Hyp2 protein was to be expected. The resulting constructs are schematically depicted in Figure 2a. Sequences of the plasmids are shown in Figure S2. The cassettes described above were excised from pUC19 by restriction with EcoRI and KpnI, and the DNA was used to transform *S. cerevisiae* strains, and histidine prototrophic transformants were selected. Correct replacement of the endogenous *HYP2* locus by the *His3MX6-*marked conditional alleles was verified by PCR and phenotypic analyses. To ascertain that its expression could not interfere with establishing the *HYP2* shutdown (*HYP2*^SD^) phenotype, the *ANB1/HYP1* gene was deleted and replaced with a *KanMX6* marker [56]. Prototrophic spore clones bearing *HA-HYP2^SD^* and *anb1∆::KanMX6*, as well as the reporter 2xMYC-*DHFR*-RFS*^OAZ1^*-2xHA, were obtained after crossing and tetrad dissection.

### 4.4. Generation of DYS1 Shutdown (SD) Strains

Conditional *DYS1* constructs (pKH13 and pKH14) were created by long-flanking overhang PCRs with the use of Ultramer primers (~190 bp) (Integrated DNA Technologies, Coralville, IA, USA) using pKH15 or pKH16 (described in the previous section), respectively, as templates. The forward Ultramer oligonucleotide primer contained a NotI site, an upstream segment of 156 bp from the 5′ untranslated region of the *DYS1* gene, and 25 Bp matching the 5′ end of the *His3MX6* marker cassette. The reverse Ultramer oligonucleotide primer contained a KpnI site, a 5′ segment of 158 bp from the *DYS1 ORF*, and 25 Bp matching to the HA tag. The resulting PCR products were cloned into the pJET1.2 vector (Thermo Fisher Scientific, Waltham, MA, USA) by blunt end ligation and were verified by sequencing. The obtained constructs are schematically depicted in Figure 2a. Sequences of the plasmids are shown in Supplementary Figure S2. The resulting cassettes described above were excised from pJET1.2 by restriction with NotI and KpnI, the DNA was used to transform *S. cerevisiae* strains, and histidine prototrophic transformants were selected. Correct replacement of the endogenous *DYS1* locus by the *His3MX6-*marked conditional alleles was verified by PCR and phenotypic analyses. Prototrophic spore clones bearing *Ub-R-HA-DYS1^SD^* and the reporters 2xMYC-*DHFR*-RFS*^OAZ1^*-2xHA or 2xMYC-*DHFR*-if-2xHA were obtained after crossing and tetrad dissection.

### 4.5. Analysis of Ribosomal Frameshift Efficiency in HYP2 and DYS1 Shutdown (SD) Strains

Yeast cells from exponentially growing cultures were harvested by centrifugation at 5000× *g* for 5 min). The pellets were shock-frozen in liquid nitrogen and stored at −80 °C. Extraction of proteins from yeast cells pellets was performed by boiling in 50 µL SDS sample buffer [67] per OD_600_ unit (corresponds to 1 mL of cells with an OD_600_ of 1) for 5 min at 99 °C. Protein analysis by SDS-polyacrylamide gel electrophoresis (SDS-PAGE) was performed with 12% PAA gels and western blotting as described [67]. Extracts from cells corresponding to one OD_600_ unit were loaded. Additionally, the PageRuler^TM^ Plus Prestained Protein Ladder (Thermo Fisher Scientific, Waltham, MA, USA) was loaded as a size marker. Transfer of proteins to nitrocellulose membranes was performed by wet blotting using boric acid transfer buffer (50 mM boric acid, 10% MeOH, pH 8.5) at 4 °C for 16 h at 20 V and 100 mA. Loading and blotting of proteins were evaluated by REVERT Total Protein Stain (LI-COR, Lincoln, NE, USA). Antibodies used for immunodetection are listed in Table S4. Membranes were scanned using an LI-COR Odyssey Imager, and the signals were quantified and analyzed with Image Studio Lite Ver 5.2 (LI-COR, Lincoln, NE, USA). To determine efficiency of ribosomal frameshifting (RFS) during decoding of the reporter mRNAs, the signals of the full-length protein resulting from RFS and of the shorter polypeptide resulting from premature termination at the internal STOP codon were quantified and together defined as 100% reporter in a given sample. The amount of the full-length reporter was calculated relative to this total amount and defined as % RFS. Statistical analysis and graph generation was done using GraphPad Prism software (v8.4.3, 2020; GraphPad Software, San Diego, CA, USA).

### 4.6. Effect of DFMO-Induced Polyamine Depletion on Ribosomal Frameshift Efficiency

To determine the effect of polyamine depletion on RFS, the ODC inhibitor DFMO was applied to synthetic dextrose (=glucose) (SD) cultures at 5 mM final concentration. The inhibitor was added to exponentially growing prototrophic wild-type cells expressing either the RFS or the in-frame control. Cells were grown in the presence of the inhibitor for up to 24 h. In order to keep cells growing without limiting nutrients, the cultures were kept below an OD_600nm_ of 1 by diluting them in fresh DFMO-containing medium at each intermittent time point.

## Data Availability

Data is contained within the article and Supplementary material.

## Supplementary Materials

The following supporting information can be downloaded at: https://www.mdpi.com/article/10.3390/ijms232112972/s1.

## References

[1] Tabor C.W., Tabor H. (1984). Polyamines. Annu. Rev. Biochem..

[2] Michael A.J. (2016). Biosynthesis of polyamines and polyamine-containing molecules. Biochem. J..

[3] Schwartz B., Hittelman A., Daneshvar L., Basu H.S., Marton L.J., Feuerstein B.G. (1995). A new model for disruption of the ornithine decarboxylase gene, SPE1, in Saccharomyces cerevisiae exhibits growth arrest and genetic instability at the MAT locus. Biochem. J..

[4] Wallace H.M., Fraser A.V., Hughes A. (2003). A perspective of polyamine metabolism. Biochem. J..

[5] Minois N., Carmona-Gutierrez D., Madeo F. (2011). Polyamines in aging and disease. Aging (Albany NY).

[6] Palanimurugan R., Kurian L., Hegde V., Hofmann K., Dohmen R.J., Ito K. (2014). Co-translational Polyamine Sensing by Nascent ODC Antizyme. Regulatory Nascent Polypeptides.

[7] Igarashi K., Kashiwagi K. (2019). The functional role of polyamines in eukaryotic cells. Int. J. Biochem. Cell Biol..

[8] Eisenberg T., Knauer H., Schauer A., Buttner S., Ruckenstuhl C., Carmona-Gutierrez D., Ring J., Schroeder S., Magnes C., Antonacci L. (2009). Induction of autophagy by spermidine promotes longevity. Nat. Cell Biol..

[9] Zhang H., Alsaleh G., Feltham J., Sun Y., Napolitano G., Riffelmacher T., Charles P., Frau L., Hublitz P., Yu Z. (2019). Polyamines Control eIF5A Hypusination, TFEB Translation, and Autophagy to Reverse B Cell Senescence. Mol. Cell.

[10] Nowotarski S.L., Woster P.M., Casero R.A. (2013). Polyamines and cancer: Implications for chemotherapy and chemoprevention. Expert Rev. Mol. Med..

[11] Pegg A.E. (2009). S-Adenosylmethionine decarboxylase. Essays Biochem..

[12] Soda K. (2018). Polyamine Metabolism and Gene Methylation in Conjunction with One-Carbon Metabolism. Int. J. Mol. Sci..

[13] Kern A.D., Oliveira M.A., Coffino P., Hackert M.L. (1999). Structure of mammalian ornithine decarboxylase at 1.6 A resolution: Stereochemical implications of PLP-dependent amino acid decarboxylases. Struct. Fold Des..

[14] Palanimurugan R., Scheel H., Hofmann K., Dohmen R.J. (2004). Polyamines regulate their synthesis by inducing expression and blocking degradation of ODC antizyme. Embo. J..

[15] Kahana C. (2018). The antizyme family for regulating polyamines. J. Biol. Chem..

[16] Coffino P. (2001). Regulation of cellular polyamines by antizyme. Nat. Rev. Mol. Cell Biol..

[17] Gödderz D., Schäfer E., Palanimurugan R., Dohmen R.J. (2011). The N-terminal unstructured domain of yeast ODC functions as a transplantable and replaceable ubiquitin-independent degron. J. Mol. Biol..

[18] Murakami Y., Matsufuji S., Kameji T., Hayashi S., Igarashi K., Tamura T., Tanaka K., Ichihara A. (1992). Ornithine decarboxylase is degraded by the 26S proteasome without ubiquitination. Nature.

[19] Kurian L., Palanimurugan R., Gödderz D., Dohmen R.J. (2011). Polyamine sensing by nascent ornithine decarboxylase antizyme stimulates decoding of its mRNA. Nature.

[20] Matsufuji S., Matsufuji T., Miyazaki Y., Murakami Y., Atkins J.F., Gesteland R.F., Hayashi S. (1995). Autoregulatory frameshifting in decoding mammalian ornithine decarboxylase antizyme. Cell.

[21] Ivanov I.P., Atkins J.F. (2007). Ribosomal frameshifting in decoding antizyme mRNAs from yeast and protists to humans: Close to 300 cases reveal remarkable diversity despite underlying conservation. Nucleic Acids Res..

[22] Petros L.M., Howard M.T., Gesteland R.F., Atkins J.F. (2005). Polyamine sensing during antizyme mRNA programmed frameshifting. Biochem. Biophys. Res. Commun..

[23] Howard M.T., Shirts B.H., Zhou J., Carlson C.L., Matsufuji S., Gesteland R.F., Weeks R.S., Atkins J.F. (2001). Cell culture analysis of the regulatory frameshift event required for the expression of mammalian antizymes. Genes Cells.

[24] Dever T.E., Gutierrez E., Shin B.S. (2014). The hypusine-containing translation factor eIF5A. Crit. Rev. Biochem. Mol. Biol..

[25] Saini P., Eyler D.E., Green R., Dever T.E. (2009). Hypusine-containing protein eIF5A promotes translation elongation. Nature.

[26] Schuller A.P., Wu C.C., Dever T.E., Buskirk A.R., Green R. (2017). eIF5A Functions Globally in Translation Elongation and Termination. Mol. Cell.

[27] Pelechano V., Alepuz P. (2017). eIF5A facilitates translation termination globally and promotes the elongation of many non polyproline-specific tripeptide sequences. Nucleic Acids Res..

[28] Manjunath H., Zhang H., Rehfeld F., Han J., Chang T.C., Mendell J.T. (2019). Suppression of Ribosomal Pausing by eIF5A Is Necessary to Maintain the Fidelity of Start Codon Selection. Cell Rep..

[29] Schwelberger H.G., Kang H.A., Hershey J.W. (1993). Translation initiation factor eIF-5A expressed from either of two yeast genes or from human cDNA. Functional identity under aerobic and anaerobic conditions. J. Biol. Chem..

[30] Schnier J., Schwelberger H.G., Smit-McBride Z., Kang H.A., Hershey J.W. (1991). Translation initiation factor 5A and its hypusine modification are essential for cell viability in the yeast Saccharomyces cerevisiae. Mol. Cell. Biol..

[31] Gutierrez E., Shin B.S., Woolstenhulme C.J., Kim J.R., Saini P., Buskirk A.R., Dever T.E. (2013). eIF5A Promotes Translation of Polyproline Motifs. Mol. Cell.

[32] Schrader R., Young C., Kozian D., Hoffmann R., Lottspeich F. (2006). Temperature-sensitive eIF5A mutant accumulates transcripts targeted to the nonsense-mediated decay pathway. J. Biol. Chem..

[33] Park M.H., Wolff E.C., Folk J.E. (1993). Hypusine: Its post-translational formation in eukaryotic initiation factor 5A and its potential role in cellular regulation. Biofactors.

[34] Chattopadhyay M.K., Park M.H., Tabor H. (2008). Hypusine modification for growth is the major function of spermidine in Saccharomyces cerevisiae polyamine auxotrophs grown in limiting spermidine. Proc. Natl. Acad. Sci. USA.

[35] Park M.H., Wolff E.C. (2018). Hypusine, a polyamine-derived amino acid critical for eukaryotic translation. J. Biol. Chem..

[36] Galvao F.C., Rossi D., Silveira Wda S., Valentini S.R., Zanelli C.F. (2013). The deoxyhypusine synthase mutant dys1-1 reveals the association of eIF5A and Asc1 with cell wall integrity. PLoS ONE.

[37] Park M.H. (2006). The post-translational synthesis of a polyamine-derived amino acid, hypusine, in the eukaryotic translation initiation factor 5A (eIF5A). J. Biochem..

[38] Thompson G.M., Cano V.S., Valentini S.R. (2003). Mapping eIF5A binding sites for Dys1 and Lia1: In vivo evidence for regulation of eIF5A hypusination. FEBS Lett..

[39] Jao D.L., Chen K.Y. (2006). Tandem affinity purification revealed the hypusine-dependent binding of eukaryotic initiation factor 5A to the translating 80S ribosomal complex. J. Cell. Biochem..

[40] Sasaki K., Abid M.R., Miyazaki M. (1996). Deoxyhypusine synthase gene is essential for cell viability in the yeast Saccharomyces cerevisiae. FEBS Lett..

[41] Almeida O.P., Toledo T.R., Rossi D., Rossetto D.D., Watanabe T.F., Galvao F.C., Medeiros A.I., Zanelli C.F., Valentini S.R. (2013). Hypusine Modification Of The Ribosome-Binding Protein Eif5a, A Target For New Anti-Inflammatory Drugs: Understanding The Action Of The Inhibitor GC7 on a Murine Macrophage Cell Line. Curr. Pharm. Des..

[42] Lee Y.B., Park M.H., Folk J.E. (1995). Diamine and triamine analogs and derivatives as inhibitors of deoxyhypusine synthase: Synthesis and biological activity. J. Med. Chem..

[43] Schmidt C., Becker T., Heuer A., Braunger K., Shanmuganathan V., Pech M., Berninghausen O., Wilson D.N., Beckmann R. (2016). Structure of the hypusinylated eukaryotic translation factor eIF-5A bound to the ribosome. Nucleic Acids Res..

[44] Nakanishi S., Cleveland J.L. (2016). Targeting the polyamine-hypusine circuit for the prevention and treatment of cancer. Amino Acids.

[45] Mathews M.B., Hershey J.W. (2015). The translation factor eIF5A and human cancer. Biochim. Biophys. Acta.

[46] Scuoppo C., Miething C., Lindqvist L., Reyes J., Ruse C., Appelmann I., Yoon S., Krasnitz A., Teruya-Feldstein J., Pappin D. (2012). A tumour suppressor network relying on the polyamine-hypusine axis. Nature.

[47] Liang Y., Piao C., Beuschel C.B., Toppe D., Kollipara L., Bogdanow B., Maglione M., Lutzkendorf J., See J.C.K., Huang S. (2021). eIF5A hypusination, boosted by dietary spermidine, protects from premature brain aging and mitochondrial dysfunction. Cell Rep..

[48] Madeo F., Eisenberg T., Buttner S., Ruckenstuhl C., Kroemer G. (2010). Spermidine: A novel autophagy inducer and longevity elixir. Autophagy.

[49] Kim H.I., Schultz C.R., Chandramouli G.V.R., Geerts D., Risinger J.I., Bachmann A.S. (2022). Pharmacological targeting of polyamine and hypusine biosynthesis reduces tumour activity of endometrial cancer. J. Drug Target..

[50] Holbert C.E., Cullen M.T., Casero R.A., Stewart T.M. (2022). Polyamines in cancer: Integrating organismal metabolism and antitumour immunity. Nat. Rev. Cancer.

[51] Dohmen R.J., Wu P., Varshavsky A. (1994). Heat-inducible degron: A method for constructing temperature-sensitive mutants. Science.

[52] Faden F., Ramezani T., Mielke S., Almudi I., Nairz K., Froehlich M.S., Höckendorff J., Brandt W., Hoehenwarter W., Dohmen R.J. (2016). Phenotypes on demand via switchable target protein degradation in multicellular organisms. Nat. Commun..

[53] Stark M.J.R., Stansfield I., Stark M.J.R. (2007). Studying Essential Genes: Generating and Using Promoter Fusions and Conditional Alleles.

[54] Kötter P., Weigand J.E., Meyer B., Entian K.D., Suess B. (2009). A fast and efficient translational control system for conditional expression of yeast genes. Nucleic Acids Res..

[55] Varshavsky A. (2019). N-degron and C-degron pathways of protein degradation. Proc. Natl. Acad. Sci. USA.

[56] Longtine M.S., McKenzie A., Demarini D.J., Shah N.G., Wach A., Brachat A., Philippsen P., Pringle J.R. (1998). Additional modules for versatile and economical PCR-based gene deletion and modification in Saccharomyces cerevisiae. Yeast.

[57] Ramos P.C., Höckendorff J., Johnson E.S., Varshavsky A., Dohmen R.J. (1998). Ump1p is required for proper maturation of the 20S proteasome and becomes its substrate upon completion of the assembly. Cell.

[58] Puleston D.J., Buck M.D., Klein Geltink R.I., Kyle R.L., Caputa G., O’Sullivan D., Cameron A.M., Castoldi A., Musa Y., Kabat A.M. (2019). Polyamines and eIF5A Hypusination Modulate Mitochondrial Respiration and Macrophage Activation. Cell Metab..

[59] Dever T.E., Ivanov I.P. (2018). Roles of polyamines in translation. J. Biol. Chem..

[60] Beenukumar R.R., Gödderz D., Palanimurugan R., Dohmen R.J. (2015). Polyamines directly promote antizyme-mediated degradation of ornithine-decarboxylase by the proteasome. Microb. Cell.

[61] Vindu A., Shin B.S., Choi K., Christenson E.T., Ivanov I.P., Cao C., Banerjee A., Dever T.E. (2021). Translational autoregulation of the S. cerevisiae high-affinity polyamine transporter Hol1. Mol. Cell.

[62] Schultz C.R., Geerts D., Mooney M., El-Khawaja R., Koster J., Bachmann A.S. (2018). Synergistic drug combination GC7/DFMO suppresses hypusine/spermidine-dependent eIF5A activation and induces apoptotic cell death in neuroblastoma. Biochem. J..

[63] Heinemeyer W., Gruhler A., Mohrle V., Mahe Y., Wolf D.H. (1993). PRE2, highly homologous to the human major histocompatibility complex-linked RING10 gene, codes for a yeast proteasome subunit necessary for chrymotryptic activity and degradation of ubiquitinated proteins. J. Biol. Chem..

[64] Sikorski R.S., Hieter P. (1989). A system of shuttle vectors and yeast host strains designed for efficient manipulation of DNA in Saccharomyces cerevisiae. Genetics.

[65] Vieira J., Messing J. (1991). New pUC-derived cloning vectors with different selectable markers and DNA replication origins. Gene.

[66] Yanisch-Perron C., Vieira J., Messing J. (1985). Improved M13 phage cloning vectors and host strains: Nucleotide sequences of the M13mp18 and pUC19 vectors. Gene.

[67] Pabst S., Döring L.M., Petreska N., Dohmen R.J. (2019). Methods to study SUMO dynamics in yeast. Methods Enzymol..

