# Supplementary material for: Hypusinated eIF5A Promotes Ribosomal Frameshifting during Decoding of ODC Antizyme mRNA in Saccharomyces cerevisiae
Source: Int J Mol Sci. 2022 Oct 26;23(21):12972. doi: 10.3390/ijms232112972 (PMC9656687; doi:10.3390/ijms232112972)
Supplement: Supplementary file 1 [file ijms-23-12972-s001.zip › ijms-1976429-supplementary.pdf]

Halwas, K., Döring, L.-D., Oehlert, F.V. and Dohmen, R.J. Supplementary Material

Material includes:

Figure S1: Western blot analyses for RFS as well as in-frame reporters, and total protein staining relating to Figure 4

Figure S2: Plasmid sequences

Table S1: Quantification of anti-Myc western blot signals of blot shown in Fig. 4

Table S2: S. cerevisiae strains used in this study

Table S3: Plasmids used in this study

Table S4: Antibodies used in this study

Figure S1

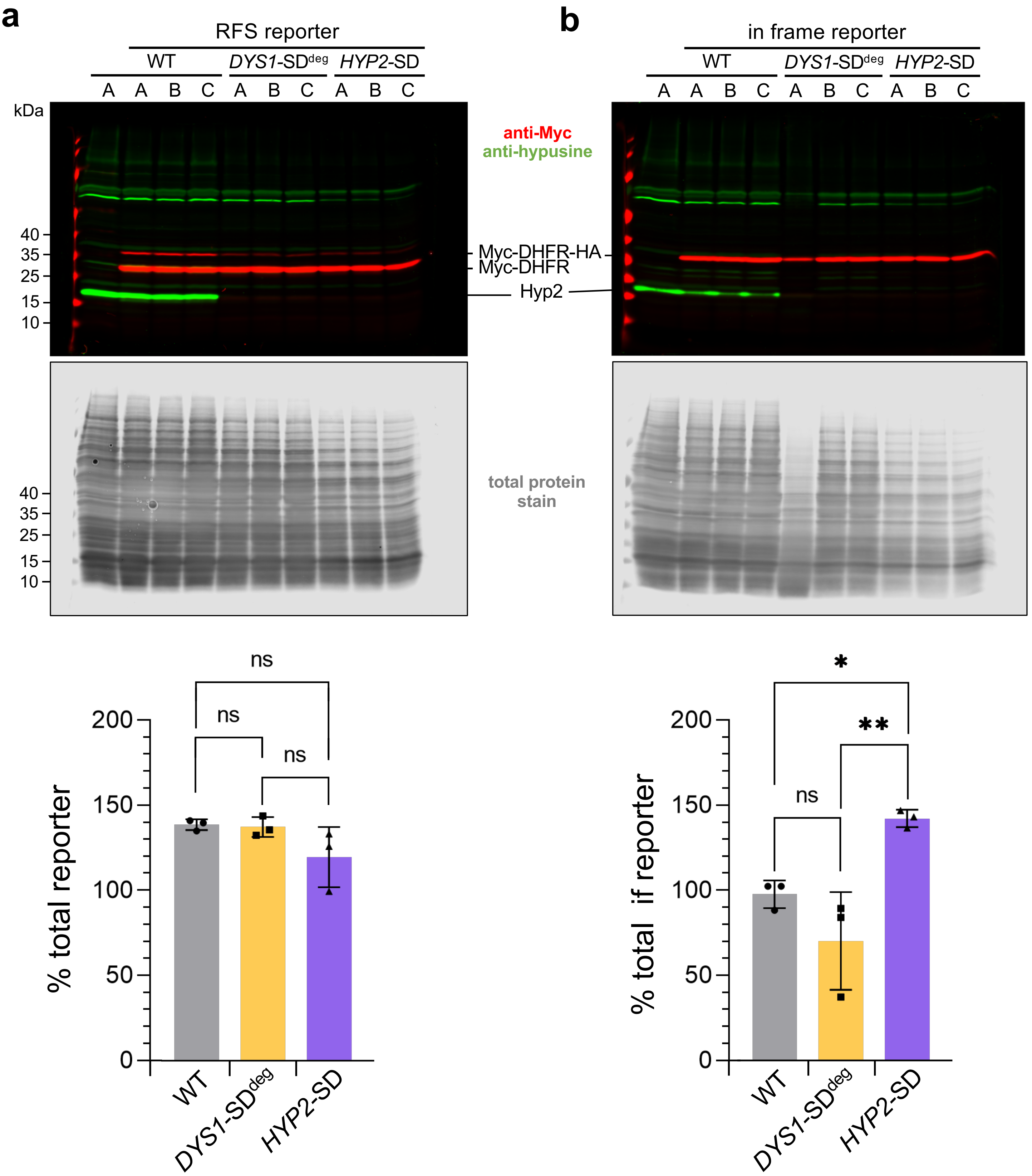
a
RFS reporter
WT
DYS1-SDdeg
HYP2-SD
A A B C A B C A B C
kDa
40
35
25
15
10
anti-Myc
anti-hypusine
Myc-DHFR-HA
Myc-DHFR
Hyp2
total protein stain
b
in frame reporter
WT
DYS1-SDdeg
HYP2-SD
A A B C A B C A B C
% total reporter
ns
ns
ns
200
150
100
50
0
WT
DYS1-SDdeg
HYP2-SD
% total if reporter
*
**
ns

Legend to Figure S1:

Western blot analyses for RFS as well as in-frame reporters, and total protein staining relating to Figure 4. (**a**) Shown are the additional data for the same blots shown in figure 4a, in which wild type (wt), *HYP2*-SD, and *DYS1*-SD^{deg} expressing the RFS reporter were analyzed. The top panel shows the full scan of the blot after anti-MYC and anti-hypusine detection. The lower panel shows the total protein stain of the same blot. Below the quantification of the total reporter signal (signals for both bands added together) after normalization to total protein signals is shown. Means, standard deviations and significance were calculated as in figure 4. (b) same as in (a) but for cells expressing the in frame (if) reporter. For the quantification, the signals of one apparently underloaded lane with one of the *DYS1*-SD^{deg} spore clones was not considered.

Figure S2: Plasmid sequences

pFO1/2 pUC21-NotI-P_{URA3}-*URA3*-P_{ADH1}-2xMyc-DHFR-RFS_{OAZ1}(t)/if-2xHA-T_{CYC1}-T_{URA3}-NotI

agcgcccaatacgcaaaccgcctctccccgcgcgttggccgattcattaatgcagctggcacgacaggtttcccgactggaaagcgggcagtgagcgc
aacgcaattaatgtgagttagctcactcattaggcaccccaggctttacactttatgcttccggctcgtatgttgtgtggaattgtgagcggataa
caatttcacacaggaaacagctatgaccatgattacgccactagtccgaggcCTCGAGGCGGCCGCAGAAAAGGATTAAAGATGCTAAGAGATAGTGA
TGATATTTCATAAATAATGTAATTCTATATATGTTAATTACCTTTTTTGCGAGGCATATTTATGGTGAAGGATAAGTTTTGACCATCAAAGAAGGTTA
ATGTGGCTGTGGTTTCAGGGTCCATAAAGCTTTTCAATTCATCtttttttttttttgttcttttttttgattccggtttctttgaaatttttttGATTC
GGTAATCTCCGAGCAGAAGGAAGAACGAAGGAAGGAGCACAGACTTAGATTGGTATATATACGCATATGTGGTGTTGAAGAAACATGAAATTGCCCAG
TATTCTTAACCCAACTGCACAGAACAAAAACCTGCAGGAAACGAAGATAAATCATGTCGAAAGCTACATATAAGGAACGTGCTGCTACTCATCCTAGT
CCTGTTGCTGCCAAGCTATTTAATATCATGCACGAAAAGCAAACAAACTTGTGTGCTTCATTGGATGTTCGTACCACCAAGGAATTACTGGAGTTAGT
TGAAGCATTAGGTCCCAAAATTTGTTTACTAAAAACACATGTGGATATCTTGACTGATTTTTCCATGGAGGGCACAGTTAAGCCGCTAAAGGCATTAT
CCGCCAAGTACAATTTTTTACTCTTCGAAGACAGAAAATTTGCTGACATTGGTAATACAGTCAAATTGCAGTACTCTGCGGGTGTATACAGAATAGCA
GAATGGGCAGACATTACGAATGCACACGGTGTGGTGGGCCCAGGTATTGTTAGCGGTTTGAAGCAGGCGGCGGAAGAAGTAACAAAGGAACCTAGAGG
CCTTTTGATGTTAGCAGAATTGTCATGCAAGGGCTCCCTAGCTACTGGAGAATATACTAAGGGTACTGTTGACATTGCGAAGAGCGACAAAGATTTTG
TTATCGGCTTTATTGCTCAAAGAGACATGGGTGGAAGAGATGAAGGTTACGATTGGTTGATTATGACACCCGGTGTGGGTTTAGATGACAAGGGAGAC
GCATTGGGTCAACAGTATAGAACCGTGGATGATGTGGTCTCTACAGGATCTGACATTATTATTGTTGGAAGAGGACTATTTGCAAAGGGAAGGGATGC
TAAGGTAGAGGGTGAACGTTACAGAAAAGCAGGCTGGGAAGCATATTTGAGAAGATGCGGCCAGCAAAACTAAGGATCCACCGGTGAGCTCAAGGTGA
GACGCGCATAACCGCTAGAGTACTTTGAAGAGGAAACAGCAATAGGGTTGCTACCAGTATAAATAGACAGGTACATACAACACTGGAAATGGTTGTCT
GTTTGAGTACGCTTTCAATTCATTTGGGTGTGCACTTTATTATGTTACAATATGGAAGGGAACTTTACACTTCTCCTATGCACATATATTAATTAAAG
TCCAATGCTAGTAGAGAAGGGGGGTAACACCCCTCCGCGCTCTTTTCCGATTTTTTTCTAAACCGTGGAATATTTCGGATATCCTTTTGTTGTTTCCG
GGTGTACAATATGGACTTCCTCTTTTCTGGCAACCAAACCCATACATCGGGATTCCTATAATACCTTCGTTGGTCTCCCTAACATGTAGGTGGCGGAG
GGGAGATATACAATAGAACAGATACCAGACAAGACATAATGGGCTAAACAAGACTACACCAATTACACTGCCTCATTGATGGTGGTACATAACGAACT
AATACTGTAGCCCTAGACTTGATAGCCATCATCATATCGAAGTTTCACTACCCTTTTTCCATTTGCCATCTATTGAAGTAATAATAGGCGCATGCAAC
TTCTTTTCTTTTTTTTTCTTTTCTCTCTCCCCCGTTGTTGTCTCACCATATCCGCAATGACAAAAAAATGATGGAAGACACTAAAGGAAAAAATTAAC
GACAAAGACAGCACCAACAGATGTCGTTGTTCCAGAGCTGATGAGGGGTATCTCGAAGCACACGAAACTTTTTCCTTCCTTCATTCACGCACACTACT
CTCTAATGAGCAACGGTATACGGCCTTCCTTCCAGTTACTTGAATTTGAAATAAAAAAAAGTTTGCTGTCTTGCTATCAAGTATAAATAGACCTGCAA
TTATTAATCTTTTGTTTCCTCGTCATTGTTCTCGTTCCCTTTCTTCCTTGTTTCTTTTTCTGCACAATATTTCAAGCTATACCAAGCATACAATCAAC
TATCTCATATACAAGATCTatggagcaaaagctcatttctgaagaggacttgaatggagaacagaaattgatcagtgaggaagacctcggtgAATTCa
tggttcgaccattgaactgcatcgtcgccgtgtcccaaaatatggggattggcaagaacggagaccgaccctggcctccgctcaggaacgagttcaag
tacttccaaagaatgaccacaacctcttcagtggaaggtaaacagaatctggtgattatgggtaggaaaacctggttctccattcctgagaagaatcg
acctttaaaggacagaattaatatagttctcagtagagaactcaaagaaccaccacgaggagctcattttcttgccaaaagtttggatgatgccttaa
gacttattgaacaaccggaattggcaagtaaagtagacatggtttggatagtcggaggcagttctgtttaccaggaagccatgaatcaaccaggccac
ctcagactctttgtgacaaggatcatgcaggaatttgaaagtgacacgtttttcccagaaattgatttggggaaatataaacttctcccagaataccc
aggcgtcctctctgaggtccaggaggaaaaaggcatcaagtataagtttgaagtctacgagaagaaagacCTGTCGACTGGGGGATTTAAGGATTGGT
GCGCGtGACATCCCTCTAGACGTTCCACCAGGATCCGGGTACCCATACGATGTTCCAGATTACGCTATGGGCGGATACCCATATGACGTACCTGACTA
TGCAGTCTAGAGGATCGATCCCCGGGTACGTACCAAGATGGCCTTTGGTGGGTTGAAGAAGGAAAAAGACAGAAACGACTTAATTACCTACTTGAAAA
AAGCCTGTGAGTAAACAGGCCCCTTTTCCTTTGTCGATATCATGTAATTAGTTATGTCACGCTTACATTCACGCCCTCCCCCCACATCCGCTCTAACC
GAAAAGGAAGGAGTTAGACAACCTGAAGTCTAGGTCCCTATTTATTTTTTTATAGTTATGTTAGTATTAAGAACGTTATTTATATTTCAAATTTTTCT
TTTTTTTCTGTACAGACGCGTGTACGCATGTAACATTATACTGAAAACCTTGCTTGAGAAGGTTTTGGGACGCTCGAAGGCTTTAATTTGCCTGCAGG
CATGCAGATCTGCTAGCGTCGACAAAACTGTATTATAAGTAAATGCATGTATACTAAACTCACAAATTAGAGCTTCAATTTAATTATATCAGTTATTA
CCCGGGAATCTCGGTCGTAATGATTTCTATAATGACGaaaaaaaaaaaaTTGGAAAGAAAAAGCTTCATGGCCTTTATAAAAAGGAACTATCCAATAC
CTCGCCAGAACCAAGTAACAGTATTTTACGGGGCACAAATCAAGAACAATAAGACAGGACTGTAAAGATGGACGCATTGAACTCCAAAGAACAACAAG
AGTTCCAAAAAGTAGTGGAACAAAAGCAAATGAAGGATTTCATGCGTTTGTACTCTAATCTGGTAGAAAGATGTTTCACAGACTGTGTCAATGACTTC
ACAACATCAAAGCTAACCAATAAGGAACAAACATGCATCATGAAGTGCTCAGAAAAGTTCTTGAAGCATAGCGAACGTGTAGGGCAGCGTTTCCAAGA
ACAAAACGCTGCCTTGGGACAAGGCTTGGGCCGATAAGGTGTACGCGGCCGCTCTAGAactagtggatcgatccccaattcgccctatagtgagtcgt
attacaattcactggccgtcgttttacaacgtcgtgactgggaaaaccctggcgttacccaacttaatcgccttgcagcacatccccctttcgccagc
tggcgtaatagcgaagaggcccgcaccgatcgcccttcccaacagttgcgcagcctgaatggcgaatggcgcctgatgcggtattttctccttacgca
tctgtgcggtatttcacaccgcatacgtcaaagcaaccatagtacgcgccctgtagcggcgcattaagcgcggcgggtgtggtggttacgcgcagcgt
gaccgctacacttgccagcgccTtagcgcccgctcctttcgctttcttcccttcctttctcgccacgttcgccggctttccccgtcaagctctaaatc
ggggtgggccatcgccctgatagacggtttttcgccctttgacgttggagtccacgttctttaatagtggactcttgttccaaactggaacaacactc
aacTctatctcgggctattcttttgatttataagggattttgccgatttcggTctattggttaaaaaatgagctgatttaacaaaaatttaacgcgaa
ttttaacaaaatattaacgtttacaattttatggtgcactctcagtacaatctgctctgatgccgcatagttaagccagccccgacacccgccaacac
ccgctgacgcgccctgacgggcttgtctgctcccggcatccgcttacagacaagctgtgaccgtctccgggagctgcatgtgtcagaggttttcaccg
tcatcaccgaaacgcgcgagacgaaagggcctcgtgatacgcctatttttataggttaatgtcatgataataatggtttcttagacgtcaggtggcac
ttttcggggaaatgtgcgcggaacccctatttgtttatttttctaaatacattcaaatatgtatccgctcatgagacaataaccctgataaatgcttc
aataatattgaaaaaggaagagtatgagtattcaacatttccgtgtcgcccttattcccttttttgcggcattttgccttcctgtttttgctcaccca
gaaacgctggtgaaagtaaaagatgctgaagatcagttgggtgcacgagtgggttacatcgaactggatctcaacagcggtaagatccttgagagttt
tcgccccgaagaacgttttccaatgatgagcacttttaaagttctgctatgtggcgcggtattatcccgtattgacgccgggcaagagcaactcggtc
gccgcatacactattctcagaatgacttggttgagtactcaccagtcacagaaaagcatcttacggatggcatgacagtaagagaattatgcagtgct
gccataaccatgagtgataacactgcggccaacttacttctgacaacgatcggaggaccgaaggagctaaccgcttttttgcacaacatgggggatca
tgtaactcgccttgatcgttgggaaccggagctgaatgaagccataccaaacgacgagcgtgacaccacgatgcctgtagcaatggcaacaacgttgc
gcaaactattaactggcgaactacttactctagcttcccggcaacaattaatagactggatggaggcggataaagttgcaggaccacttctgcgctcg
gcccttccggctggctggtttattgctgataaatctggagccggtgagcgtgggtctcgcggtatcattgcagcactggggccagatggtaagccctc
ccgtatcgtagttatctacacgacggggagtcaggcaactatggatgaacgaaatagacagatcgctgagataggtgcctcactgattaagcattggt
aactgtcagaccaagtttactcatatatactttagattgatttaaaacttcatttttaatttaaaaggatctaggtgaagatcctttttgataatctc
atgaccaaaatcccttaacgtgagttttcgttccactgagcgtcagaccccgtagaaaagatcaaaggatcttcttgagatcctttttttctgcgcgt
aatctgctgcttgcaaacaaaaaaaccaccgctaccagcggtggtttgtttgccggatcaagagctaccaactctttttccgaaggtaactggcttca
gcagagcgcagataccaaatactgtccttctagtgtagccgtagttaggccaccacttcaagaactctgtagcaccgcctacatacctcgctctgcta
atcctgttaccagtggctgctgccagtggcgataagtcgtgtcttaccgggttggactcaagacgatagttaccggataaggcgcagcggtcgggctg
aacggggggttcgtgcacacagcccagcttggagcgaacgacctacaccgaactgagatacctacagcgtgagcattgagaaagcgccacgcttcccg
aagggagaaaggcggacaggtatccggtaagcggcagggtcggaacaggagagcgcacgagggagcttccagggggaaacgcctggtatctttatagt
cctgtcgggtttcgccacctctgacttgagcgtcgatttttgtgatgctcgtcaggggggcggagcctatggaaaaacgccagcaacgcggccttttt
acggttcctggccttttgctggccttttgctcacatgttctttcctgcgttatcccctgattctgtggataaccgtattaccgcctttgagtgagctg
ataccgctcgccgcagccgaacgaccgagcgcagcgagtcagtgagcgaggaagcggaag

pKH13 [pJET1.2-P_{DYS1}-P_{TEF}-*his5*-T_{TEF}-P_{GAL1}-tc3-Ub-**R**-HA-ORF_{DYS1}]

GCCCCTGCAGCCGAATTATATTATTTTTGCCAAATAATTTTTAACAAAAGCTCTGAAGTCTTCTTCATTTAAATTCTTAGATGATACTTCATCTGGAA
AATTGTCCCAATTAGTAGCATCACGCTGTGAGTAAGTTCTAAACCATTTTTTTATTGTTGTATTATCTCTAATCTTACTACTCGATGAGTTTTCGGTA
TTATCTCTATTTTTAACTTGGAGCAGGTTCCATTCATTGTTTTTTTCATCATAGTGAATAAAATCAACTGCTTTAACACTTGTGCCTGAACACCATAT
CCATCCGGCGTAATACGACTCACTATAGGGAGAGCGGCCGCCAGATCTTCCGGATGGCTCGAGTTTTTCAGCAAGATGCAT**GCGGC**CGCATTACGGGA
AGAACTGAAAAAAAGGTGGAAAAAAAAAATAAAAAGTAAAGTGAAAAATTAAATGTAATGTATATTTCCATTTTGACGCAAAGTACTTCCCTCTATCA
GTTTTGTATCTAACTGCAGAGTAAAGTCGTGTTTTGCTTAGTTAACGAATTCGTTTAGCTTGCCTCGTCCCCGCCGGGTCACCCGGCCAGCGACATGG
AGGCCCAGAATACCCTCCTTGACAGTCTTGACGTGCGCAGCTCAGGGGCATGATGTGACTGTCGCCCGTACATTTAGCCCATACATCCCCATGTATAA
TCATTTGCATCCATACATTTTGATGGCCGCACGGCGCGAAGCAAAAATTACGGCTCCTCGCTGCAGACCTGCGAGCAGGGAAACGCTCCCCTCACAGA
CGCGTTGAATTGTCCCCACGCCGCGCCCCTGTAGAGAAATATAAAAGGTTAGGATTTGCCACTGAGGTTCTTCTTTCATATACTTCCTTTTAAAATCT
TGCTAGGATACAGTTCTCACATCACATCCGAACATAAACAACCATGGGTAGGAGGGCTTTTGTAGAAAGAAATACGAACGAAACGAAAATCAGCGTTG
CCATCGCTTTGGACAAAGCTCCCTTACCTGAAGAGTCGAATTTTATTGATGAACTTATAACTTCCAAGCATGCAAACCAAAAGGGAGAACAAGTAATC
CAAGTAGACACGGGAATTGGATTCTTGGATCACATGTATCATGCACTGGCTAAACATGCAGGCTGGAGCTTACGACTTTACTCAAGAGGTGATTTAAT
CATCGATGATCATCACACTGCAGAAGATACTGCTATTGCACTTGGTATTGCATTCAAGCAGGCTATGGGTAACTTTGCCGGCGTTAAAAGATTTGGAC
ATGCTTATTGTCCACTTGACGAAGCTCTTTCTAGAAGCGTAGTTGACTTGTCGGGACGGCCCTATGCTGTTATCGATTTGGGATTAAAGCGTGAAAAG
GTTGGGGAATTGTCCTGTGAAATGATCCCTCACTTACTATATTCCTTTTCGGTAGCAGCTGGAATTACTTTGCATGTTACCTGCTTATATGGTAGTAA
TGACCATCATCGTGCTGAAAGCGCTTTTAAATCTCTGGCTGTTGCCATGCGCGCGGCTACTAGTCTTACTGGAAGTTCTGAAGTCCCAAGCACGAAGG
GAGTGTTGTAAAGAGTACTGACAATAAAAAGATTCTTGTTTTCAAGAACTTGTCATTTGTATAGTTTTTTTATATTGTAGTTGTTCTATTTTAATCAA
ATGTTAGCGTGATTTATATTTTTTTTCGCCTCGACATCATCTGCCCAGATGCGAAGTTAAGTGCGCAGAAAGTAATATCATGCGTCAATCGTATGTGA
ATGCTGGTCGCTATACTGCTGTCGATTCGATACTAACGCCGCCATCCAgtttaaac**GAGCTC**TAGTACGGATTAGAAGCCGCCGAGCGGGTGACAGCC
CTCCGAAGGAAGACTCTCCTCCGTGCGTCCTCGTCTTCACCGGTCGCGTTCCTGAAACGCAGATGTGCCTCGCGCCGCACTGCTCCGAACAATAAAGA
TTCTACAATACTAGCTTTTATGGTTATGAAGAGGAAAAATTGGCAGTAACCTGGCCCCACAAACCTTCAAATGAACGAATCAAATTAACAACCATAGG
ATGATAATGCGATTAGTTTTTTAGCCTTATTTCTGGGGTAATTAATCAGCGAAGCGATGATTTTTGATCTATTAACAGATATATAAATGCAAAAACTG
CATAACCACTTTAACTAATACTTTCAACATTTTCGGTTTGTATTACTTCTTATTCAAATGTAATAAAAGTATCAACAAAAAATTGTTAATATACCTCT
ATACTTTAACGTCAAGGAGAAAAAACCCCGCGGCCAATTATCTACTTAAGGCCTAAAACATACCAGATCGCCACCCGCGCTTTAATCTGGAGAGGTGA
AGAATACGACCACCTAGGCCTTAAGAACCGGTAAAACATACCAGATCGCCACCCGCGCTTTAATCTGGAGAGGTGAAGAATACGACCACCTACCGGTC
AACAACAACAACAACAACAACAACAACTCGAGGCCTAAAACATACCAGATCGCCACCCGCGCTTTAATCTGGAGAGGTGAAGAATACGACCACCTAGG
CCTCGAGAACATATCTACCCATACGAGATCTATGcagatttcgtcaagactttgaccggtaaaaccataacattggaagttgaatcttccgatacca
tcgacaacgttaagtcgaaaattcaagacaaggaaggtatccctccagatcaacaaagattgatctttgccggtaagcagctagaagacggtagaacg
ctgtctgattacaacattcagaaggagtccaccttacatcttgtgctaaggctaagaggtggt**AGG**CACGGATCCTACCCATACGACGTCCCAGACTA
CGCTAAAGCTTCCGGGGGGATGTCCGATATCAACGAAAAACTCCCAGAGTTACTACAAGACGCTGTCTTGAAAGCATCTGTTCCTATTCCAGATGACT
TCGTTAAGGTTCAAGGTATTGATTACTCAAAGCCTGAAGCCACTAATATGAGAGCAACTGATTTAATTGAAGCTATGAA**GGTACC**cgcATCTTTCTAG
AAGATCTCCTACAATATTCTCAGCTGCCATGGAAAATCGATGTTCTTCTTTTATTCTCTCAAGATTTTCAGGCTGTATATTAAAACTTATATTAAGAA
CTATGCTAACCACCTCATCAGGAACCGTTGTAGGTGGCGTGGGTTTTCTTGGCAATCGACTCTCATGAAAACTACGAGCTAAATATTCAATATGTTCC
TCTTGACCAACTTTATTCTGCATTTTTTTTGAACGAGGTTTAGAGCAAGCTTCAGGAAACTGAGACAGGAATTTTATTAAAAATTTAAATTTTGAAGA
AAGTTCAGGGTTAATAGCATCCATTTTTTGCTTTGCAAGTTCCTCAGCATTCTTAACAAAAGACGTCTCTTTTGACATGTTTAAAGTTTAAACCTCCT
GTGTGAAATTGTTATCCGCTCACAATTCCACACATTATACGAGCCGGAAGCATAAAGTGTAAAGCCTGGGGTGCCTAATGAGTGAGCTAACTCACATT
AATTGCGTTGCGCTCACTGCCAATTGCTTTCCAGTCGGGAAACCTGTCGTGCCAGCTGCATTAATGAATCGGCCAACGCGCGGGGAGAGGCGGTTTGC
GTATTGGGCGCTCTTCCGCTTCCTCGCTCACTGACTCGCTGCGCTCGGTCGTTCGGCTGCGGCGAGCGGTATCAGCTCACTCAAAGGCGGTAATACGG
TTATCCACAGAATCAGGGGATAACGCAGGAAAGAACATGTGAGCAAAAGGCCAGCAAAAGGCCAGGAACCGTAAAAAGGCCGCGTTGCTGGCGTTTTT
CCATAGGCTCCGCCCCCCTGACGAGCATCACAAAAATCGACGCTCAAGTCAGAGGTGGCGAAACCCGACAGGACTATAAAGATACCAGGCGTTTCCCC
CTGGAAGCTCCCTCGTGCGCTCTCCTGTTCCGACCCTGCCGCTTACCGGATACCTGTCCGCCTTTCTCCCTTCGGGAAGCGTGGCGCTTTCTCATAGC
TCACGCTGTAGGTATCTCAGTTCGGTGTAGGTCGTTCGCTCCAAGCTGGGCTGTGTGCACGAACCCCCCGTTCAGCCCGACCGCTGCGCCTTATCCGG
TAACTATCGTCTTGAGTCCAACCCGGTAAGACACGACTTATCGCCACTGGCAGCAGCCACTGGTAACAGGATTAGCAGAGCGAGGTATGTAGGCGGTG
CTACAGAGTTCTTGAAGTGGTGGCCTAACTACGGCTACACTAGAAGGACAGTATTTGGTATCTGCGCTCTGCTGAAGCCAGTTACCTTCGGAAAAAGA
GTTGGTAGCTCTTGATCCGGCAAACAAACCACCGCTGGTAGCGGTGGTTTTTTTGTTTGCAAGCAGCAGATTACGCGCAGAAAAAAAGGATCTCAAGA
AGATCCTTTGATCTTTTCTACGGGGTCTGACGCTCAGTGGAACGAAAACTCACGTTAAGGGATTTTGGTCATGAGATTATCAAAAAGGATCTTCACCT
AGATCCTTTTAAATTAAAAATGAAGTTTTAAATCAATCTAAAGTATATATGAGTAAACTTGGTCTGACAGTTACCAATGCTTAATCAGTGAGGCACCT
ATCTCAGCGATCTGTCTATTTCGTTCATCCATAGTTGCCTGACTCCCCGTCGTGTAGATAACTACGATACGGGAGGGCTTACCATCTGGCCCCAGTGC
TGCAATGATACCGCGAGACCCACGCTCACCGGCTCCAGATTTATCAGCAATAAACCAGCCAGCCGGAAGGGCCGAGCGCAGAAGTGGTCCTGCAACTT
TATCCGCCTCCATCCAGTCTATTAATTGTTGCCGGGAAGCTAGAGTAAGTAGTTCGCCAGTTAATAGTTTGCGCAACGTTGTTGCCATTGCTACAGGC
ATCGTGGTGTCACGCTCGTCGTTTGGTATGGCTTCATTCAGCTCCGGTTCCCAACGATCAAGGCGAGTTACATGATCCCCCATGTTGTGCAAAAAAGC
GGTTAGCTCCTTCGGTCCTCCGATCGTTGTCAGAAGTAAGTTGGCCGCAGTGTTATCACTCATGGTTATGGCAGCACTGCATAATTCTCTTACTGTCA
TGCCATCCGTAAGATGCTTTTCTGTGACTGGTGAGTACTCAACCAAGTCATTCTGAGAATAGTGTATGCGGCGACCGAGTTGCTCTTGCCCGGCGTCA
ATACGGGATAATACCGCGCCACATAGCAGAACTTTAAAAGTGCTCATCATTGGAAAACGTTCTTCGGGGCGAAAACTCTCAAGGATCTTACCGCTGTT
GAGATCCAGTTCGATGTAACCCACTCGTGCACCCAACTGATCTTCAGCATCTTTTACTTTCACCAGCGTTTCTGGGTGAGCAAAAACAGGAAGGCAAA
ATGCCGCAAAAAAGGGAATAAGGGCGACACGGAAATGTTGAATACTCATACTCTTCCTTTTTCAATATTATTGAAGCATTTATCAGGGTTATTGTCTC
ATGAGCGGATACATATTTGAATGTATTTAGAAAAATAAACAAATAGGGGTTCCGCGCACATTTCCCCGAAAAGTGCCACCTGACGTCTAAGAAACCAT
TATTATCATGACATTAACCTATAAAAATAGGCGTATCACGAGGCC

pKH14 [pJET1.2-P_{DYS1}-P_{TEF}-*his5*-T_{TEF}-P_{GAL1}-tc3-HA-ORF_{DYS1}]

GCCCCTGCAGCCGAATTATATTATTTTTGCCAAATAATTTTTAACAAAAGCTCTGAAGTCTTCTTCATTTAAATTCTTAGATGATACTTCATCTGGAA
AATTGTCCCAATTAGTAGCATCACGCTGTGAGTAAGTTCTAAACCATTTTTTTATTGTTGTATTATCTCTAATCTTACTACTCGATGAGTTTTCGGTA
TTATCTCTATTTTTAACTTGGAGCAGGTTCCATTCATTGTTTTTTTCATCATAGTGAATAAAATCAACTGCTTTAACACTTGTGCCTGAACACCATAT
CCATCCGGCGTAATACGACTCACTATAGGGAGAGCGGCCGCCAGATCTTCCGGATGGCTCGAGTTTTTCAGCAAGATGCAT**GCGGC**CGCATTACGGGA
AGAACTGAAAAAAAGGTGGGAAAAAAAAAATAAAAAGTAAAGTGAAAAATTAAATGTAATGTATATTTCCATTTTGACGCAAAGTACTTCCCTCTATCA
GTTTTGTATCTAACTGCAGAGTAAAGTCGTGTTTTGCTTAGTTAACGAATTCGTTTAGCTTGCCTCGTCCCCGCCGGGTCACCCGGCCAGCGACATGG
AGGCCCAGAATACCCTCCTTGACAGTCTTGACGTGCGCAGCTCAGGGGCATGATGTGACTGTCGCCCGTACATTTAGCCCATACATCCCCATGTATAA
TCATTTGCATCCATACATTTTGATGGCCGCACGGCGCGAAGCAAAAATTACGGCTCCTCGCTGCAGACCTGCGAGCAGGGAAACGCTCCCCTCACAGA
CGCGTTGAATTGTCCCCACGCCGCGCCCCTGTAGAGAAATATAAAAGGTTAGGATTTGCCACTGAGGTTCTTCTTTCATATACTTCCTTTTAAAATCT
TGCTAGGATACAGTTCTCACATCACATCCGAACATAAACAACCATGGGTAGGAGGGCTTTTGTAGAAAGAAATACGAACGAAACGAAAATCAGCGTTG
CCATCGCTTTGGACAAAGCTCCCTTACCTGAAGAGTCGAATTTTATTGATGAACTTATAACTTCCAAGCATGCAAACCAAAAGGGAGAACAAGTAATC
CAAGTAGACACGGGAATTGGATTCTTGGATCACATGTATCATGCACTGGCTAAACATGCAGGCTGGAGCTTACGACTTTACTCAAGAGGTGATTTAAT
CATCGATGATCATCACACTGCAGAAGATACTGCTATTGCACTTGGTATTGCATTCAAGCAGGCTATGGGTAACTTTGCCGGCGTTAAAAGATTTGGAC
ATGCTTATTGTCCACTTGACGAAGCTCTTTCTAGAAGCGTAGTTGACTTGTCGGGACGGCCCTATGCTGTTATCGATTTGGGATTAAAGCGTGAAAAG
GTTGGGGAATTGTCCTGTGAAATGATCCCTCACTTACTATATTCCTTTTCGGTAGCAGCTGGAATTACTTTGCATGTTACCTGCTTATATGGTAGTAA
TGACCATCATCGTGCTGAAAGCGCTTTTAAATCTCTGGCTGTTGCCATGCGCGCGGCTACTAGTCTTACTGGAAGTTCTGAAGTCCCAAGCACGAAGG
GAGTGTTGTAAAGAGTACTGACAATAAAAAGATTCTTGTTTTCAAGAACTTGTCATTTGTATAGTTTTTTTATATTGTAGTTGTTCTATTTTAATCAA
ATGTTAGCGTGATTTATATTTTTTTTCGCCTCGACATCATCTGCCCAGATGCGAAGTTAAGTGCGCAGAAAGTAATATCATGCGTCAATCGTATGTGA
ATGCTGGTCGCTATACTGCTGTCGATTCGATACTAACGCCGCCATCCAgtttaaac**GAGCTC**TAGTACGGATTAGAAGCCGCCGAGCGGGTGACAGCC
CTCCGAAGGAAGACTCTCCTCCGTGCGTCCTCGTCTTCACCGGTCGCGTTCCTGAAACGCAGATGTGCCTCGCGCCGCACTGCTCCGAACAATAAAGA
TTCTACAATACTAGCTTTTATGGTTATGAAGAGGAAAAATTGGCAGTAACCTGGCCCCACAAACCTTCAAATGAACGAATCAAATTAACAACCATAGG
ATGATAATGCGATTAGTTTTTTAGCCTTATTTCTGGGGTAATTAATCAGCGAAGCGATGATTTTTGATCTATTAACAGATATATAAATGCAAAAACTG
CATAACCACTTTAACTAATACTTTCAACATTTTCGGTTTGTATTACTTCTTATTCAAATGTAATAAAAGTATCAACAAAAAATTGTTAATATACCTCT
ATACTTTAACGTCAAGGAGAAAAAACCCCGCGGCCAATTATCTACTTAAGGCCTAAAACATACCAGATCGCCACCCGCGCTTTAATCTGGAGAGGTGA
AGAATACGACCACCTAGGCCTTAAGAACCGGTAAAACATACCAGATCGCCACCCGCGCTTTAATCTGGAGAGGTGAAGAATACGACCACCTACCGGTC
AACAACAACAACAACAACAACAACAACTCGAGGCCTAAAACATACCAGATCGCCACCCGCGCTTTAATCTGGAGAGGTGAAGAATACGACCACCTAGG
CCTCGAGAACATATCTACCCATACGAGATCTATGCACGGATCCTACCCATACGACGTCCCAGACTACGCTAAAGCTTCCGGGGGATGTCCGATATCAA
CGAAAAACTCCCAGAGTTACTACAAGACGCTGTCTTGAAAGCATCTGTTCCTATTCCAGATGACTTCGTTAAGGTTCAAGGTATTGATTACTCAAAGC
CTGAAGCCACTAATATGAGAGCAACTGATTTAATTGAAGCTATGAAG**GTACC**cgcATCTTTCTAGAAGATCTCCTACAATATTCTCAGCTGCCATGGA
AAATCGATGTTCTTCTTTTATTCTCTCAAGATTTTCAGGCTGTATATTAAAACTTATATTAAGAACTATGCTAACCACCTCATCAGGAACCGTTGTAG
GTGGCGTGGGTTTTCTTGGCAATCGACTCTCATGAAAACTACGAGCTAAATATTCAATATGTTCCTCTTGACCAACTTTATTCTGCATTTTTTTTGAA
CGAGGTTTAGAGCAAGCTTCAGGAAACTGAGACAGGAATTTTATTAAAAATTTAAATTTTGAAGAAAGTTCAGGGTTAATAGCATCCATTTTTTGCTT
TGCAAGTTCCTCAGCATTCTTAACAAAAGACGTCTCTTTTGACATGTTTAAAGTTTAAACCTCCTGTGTGAAATTGTTATCCGCTCACAATTCCACAC
ATTATACGAGCCGGAAGCATAAAGTGTAAAGCCTGGGGTGCCTAATGAGTGAGCTAACTCACATTAATTGCGTTGCGCTCACTGCCAATTGCTTTCCA
GTCGGGAAACCTGTCGTGCCAGCTGCATTAATGAATCGGCCAACGCGCGGGGAGAGGCGGTTTGCGTATTGGGCGCTCTTCCGCTTCCTCGCTCACTG
ACTCGCTGCGCTCGGTCGTTCGGCTGCGGCGAGCGGTATCAGCTCACTCAAAGGCGGTAATACGGTTATCCACAGAATCAGGGGATAACGCAGGAAAG
AACATGTGAGCAAAAGGCCAGCAAAAGGCCAGGAACCGTAAAAAGGCCGCGTTGCTGGCGTTTTTCCATAGGCTCCGCCCCCCTGACGAGCATCACAA
AAATCGACGCTCAAGTCAGAGGTGGCGAAACCCGACAGGACTATAAAGATACCAGGCGTTTCCCCCTGGAAGCTCCCTCGTGCGCTCTCCTGTTCCGA
CCCTGCCGCTTACCGGATACCTGTCCGCCTTTCTCCCTTCGGGAAGCGTGGCGCTTTCTCATAGCTCACGCTGTAGGTATCTCAGTTCGGTGTAGGTC
GTTCGCTCCAAGCTGGGCTGTGTGCACGAACCCCCCGTTCAGCCCGACCGCTGCGCCTTATCCGGTAACTATCGTCTTGAGTCCAACCCGGTAAGACA
CGACTTATCGCCACTGGCAGCAGCCACTGGTAACAGGATTAGCAGAGCGAGGTATGTAGGCGGTGCTACAGAGTTCTTGAAGTGGTGGCCTAACTACG
GCTACACTAGAAGGACAGTATTTGGTATCTGCGCTCTGCTGAAGCCAGTTACCTTCGGAAAAAGAGTTGGTAGCTCTTGATCCGGCAAACAAACCACC
GCTGGTAGCGGTGGTTTTTTTGTTTGCAAGCAGCAGATTACGCGCAGAAAAAAAGGATCTCAAGAAGATCCTTTGATCTTTTCTACGGGGTCTGACGC
TCAGTGGAACGAAAACTCACGTTAAGGGATTTTGGTCATGAGATTATCAAAAAGGATCTTCACCTAGATCCTTTTAAATTAAAAATGAAGTTTTAAAT
CAATCTAAAGTATATATGAGTAAACTTGGTCTGACAGTTACCAATGCTTAATCAGTGAGGCACCTATCTCAGCGATCTGTCTATTTCGTTCATCCATA
GTTGCCTGACTCCCCGTCGTGTAGATAACTACGATACGGGAGGGCTTACCATCTGGCCCCAGTGCTGCAATGATACCGCGAGACCCACGCTCACCGGC
TCCAGATTTATCAGCAATAAACCAGCCAGCCGGAAGGGCCGAGCGCAGAAGTGGTCCTGCAACTTTATCCGCCTCCATCCAGTCTATTAATTGTTGCC
GGGAAGCTAGAGTAAGTAGTTCGCCAGTTAATAGTTTGCGCAACGTTGTTGCCATTGCTACAGGCATCGTGGTGTCACGCTCGTCGTTTGGTATGGCT
TCATTCAGCTCCGGTTCCCAACGATCAAGGCGAGTTACATGATCCCCCATGTTGTGCAAAAAAGCGGTTAGCTCCTTCGGTCCTCCGATCGTTGTCAG
AAGTAAGTTGGCCGCAGTGTTATCACTCATGGTTATGGCAGCACTGCATAATTCTCTTACTGTCATGCCATCCGTAAGATGCTTTTCTGTGACTGGTG
AGTACTCAACCAAGTCATTCTGAGAATAGTGTATGCGGCGACCGAGTTGCTCTTGCCCGGCGTCAATACGGGATAATACCGCGCCACATAGCAGAACT
TTAAAAGTGCTCATCATTGGAAAACGTTCTTCGGGGCGAAAACTCTCAAGGATCTTACCGCTGTTGAGATCCAGTTCGATGTAACCCACTCGTGCACC
CAACTGATCTTCAGCATCTTTTACTTTCACCAGCGTTTCTGGGTGAGCAAAAACAGGAAGGCAAAATGCCGCAAAAAAGGGAATAAGGGCGACACGGA
AATGTTGAATACTCATACTCTTCCTTTTTCAATATTATTGAAGCATTTATCAGGGTTATTGTCTCATGAGCGGATACATATTTGAATGTATTTAGAAA
AATAAACAAATAGGGGTTCCGCGCACATTTCCCCGAAAAGTGCCACCTGACGTCTAAGAAACCATTATTATCATGACATTAACCTATAAAAATAGGCG
TATCACGAGGCC

pKH15 [pUC19–P_{HYP2}–P_{TEF}–*his5*–T_{TEF}–P_{GAL1}–tc3–Ub–R–HA–ORF_{HYP2}–T_{HYP2}]

TCGCGCGTTTCGGTGATGACGGTGAAAACCTCTGACACATGCAGCTCCCGGAGACGGTCACAGCTTGTCTGTAAGCGGATGCCGGGAGCAGACAAGCC
CGTCAGGGCGCGTCAGCGGGTGTTGGCGGGTGTCGGGGCTGGCTTAACTATGCGGCATCAGAGCAGATTGTACTGAGAGTGCACCATATGCGGTGTGA
AATACCGCACAGATGCGTAAGGAGAAAATACCGCATCAGGCGCCATTCGCCATTCAGGCTGCGCAACTGTTGGGAAGGGCGATCGGTGCGGGCCTCTT
CGCTATTACGCCAGCTGGCGAAAGGGGGATGTGCTGCAAGGCGATTAAGTTGGGTAACGCCAGGGTTTTCCCAGTCACGACGTTGTAAAACGACGGCC
AGT**GAATTC**CCTGACCATTGGCTTCGTTCTGGTGGCATTTTCTCTGAGGGGTGAGAACAGCAAAACGGGAACGGCGACTTGGAGTAGTATAACGCTGT
AGCTGGACGCTGAGGGGCCAAGGCCGGTAAAATTCTGCAACACCCAGACCTTGCTGCAGTGATATCATCCACCCATACCCCTCGATGTATTCCGTAGC
GTTATATCGGGTCCGTGTGAGGCGACAAGGGACCTCCCGCACCGCACATACCGCAGGAGCTAAAAGAAACGCCGTCGCCCGAAAAAAAGTTTCGAAGG
TGAAGGAACAGTGGTAATAAAAATTTTTTTCGTCGTCGTCGTCGATCGGCTTTTGGCCGCGTTGATCGTCGTGTTCCACGATTATATAATGCACAAGG
TTTTTGCCATATCTTGACATTGGAATCCCTCATTTCCTAAATTCCTCTCCTTCTATCAATTGCTTCCTTTCCTTCTTTCTAACGATTCTACTTCT**aga**
tctGTTTAGCTTGCCTCGTCCCCGCCGGGTCACCCGGCCAGCGACATGGAGGCCCAGAATACCCTCCTTGACAGTCTTGACGTGCGCAGCTCAGGGGC
ATGATGTGACTGTCGCCCGTACATTTAGCCCATACATCCCCATGTATAATCATTTGCATCCATACATTTTGATGGCCGCACGGCGCGAAGCAAAAATT
ACGGCTCCTCGCTGCAGACCTGCGAGCAGGGAAACGCTCCCCTCACAGACGCGTTGAATTGTCCCCACGCCGCGCCCCTGTAGAGAAATATAAAAGGT
TAGGATTTGCCACTGAGGTTCTTCTTTCATATACTTCCTTTTAAAATCTTGCTAGGATACAGTTCTCACATCACATCCGAACATAAACAACCATGGGT
AGGAGGGCTTTTGTAGAAAGAAATACGAACGAAACGAAAATCAGCGTTGCCATCGCTTTGGACAAAGCTCCCTTACCTGAAGAGTCGAATTTTATTGA
TGAACTTATAACTTCCAAGCATGCAAACCAAAAGGGAGAACAAGTAATCCAAGTAGACACGGGAATTGGATTCTTGGATCACATGTATCATGCACTGG
CTAAACATGCAGGCTGGAGCTTACGACTTTACTCAAGAGGTGATTTAATCATCGATGATCATCACACTGCAGAAGATACTGCTATTGCACTTGGTATT
GCATTCAAGCAGGCTATGGGTAACTTTGCCGGCGTTAAAAGATTTGGACATGCTTATTGTCCACTTGACGAAGCTCTTTCTAGAAGCGTAGTTGACTT
GTCGGGACGGCCCTATGCTGTTATCGATTTGGGATTAAAGCGTGAAAAGGTTGGGGAATTGTCCTGTGAAATGATCCCTCACTTACTATATTCCTTTT
CGGTAGCAGCTGGAATTACTTTGCATGTTACCTGCTTATATGGTAGTAATGACCATCATCGTGCTGAAAGCGCTTTTAAATCTCTGGCTGTTGCCATG
CGCGCGGCTACTAGTCTTACTGGAAGTTCTGAAGTCCCAAGCACGAAGGGAGTGTTGTAAAGAGTACTGACAATAAAAAGATTCTTGTTTTCAAGAAC
TTGTCATTTGTATAGTTTTTTTATATTGTAGTTGTTCTATTTTAATCAAATGTTAGCGTGATTTATATTTTTTTTCGCCTCGACATCATCTGCCCAGA
TGCGAAGTTAAGTGCGCAGAAAGTAATATCATGCGTCAATCGTATGTGAATGCTGGTCGCTATACTGCTGTCGATTCGATACTAACGCCGCCATCCAg
tttaaac**GAGCTC**TAGTACGGATTAGAAGCCGCCGAGCGGGTGACAGCCCTCCGAAGGAAGACTCTCCTCCGTGCGTCCTCGTCTTCACCGGTCGCGT
TCCTGAAACGCAGATGTGCCTCGCGCCGCACTGCTCCGAACAATAAAGATTCTACAATACTAGCTTTTATGGTTATGAAGAGGAAAAATTGGCAGTAA
CCTGGCCCCACAAACCTTCAAATGAACGAATCAAATTAACAACCATAGGATGATAATGCGATTAGTTTTTTAGCCTTATTTCTGGGGTAATTAATCAG
CGAAGCGATGATTTTTGATCTATTAACAGATATATAAATGCAAAAACTGCATAACCACTTTAACTAATACTTTCAACATTTTCGGTTTGTATTACTTC
TTATTCAAATGTAATAAAAGTATCAACAAAAAATTGTTAATATACCTCTATACTTTAACGTCAAGGAGAAAAAACCCCGCGGCCAATTATCTACTTAA
GGCCTAAAACATACCAGATCGCCACCCGCGCTTTAATCTGGAGAGGTGAAGAATACGACCACCTAGGCCTTAAGAACCGGTAAAACATACCAGATCGC
CACCCGCGCTTTAATCTGGAGAGGTGAAGAATACGACCACCTACCGGTCAACAACAACAACAACAACAACAACAACTCGAGGCCTAAAACATACCAGA
TCGCCACCCGCGCTTTAATCTGGAGAGGTGAAGAATACGACCACCTAGGCCTCGAGAACATATCTACCCATACGAGATCTATGcagatttcgtcaag
actttgaccggtaaaaccataacattggaagttgaatcttccgataccatcgacaacgttaagtcgaaaattcaagacaaggaaggtatccctccaga
tcaacaaagattgatctttgccggtaagcagctagaagacggtagaacgctgtctgattacaacattcagaaggagtccaccttacatcttgtgctaa
ggctaagaggtggtAGGCACGGATCCTACCCATACGACGTCCCAGACTACGCTAAAGCTTCCGGGGGGATGTCTGACGAAGAACATACCTTTGAAACT
GCTGACGCTGGTTCCTCCGCCACCTACCCAATGCAATGTTCTGCCTTGAGAAAGAACGGTTTCGTTGTCATCAAGAGTAGACCATGTAAGATTGTCGA
CATGTCCACTTCTAAGACTGGTAAGCACGGTCACGCTAAAGTCCATTTGGTTGCCATTGATATCTTCACTGGTAAGAAGTTGGAAGATTTGTCTCCAT
CTACTCACAACATGGAAGTTCCAGTTGTCAAGAGAAACGAATACCAATTGTTGGACATTGATGACGGTTTCTTGTCTTTGATGAACATGGACGGTGAC
ACTAAGGATGATGTCAAGGCTCCAGAAGGTGAATTGGGTGACAGTTTGCAAACTGCTTTTGATGAAGGTAAGGACTTGATGGTTACCATCATCTCCGC
TATGGGTGAAGAAGCCGCCATCTCCTTCAAGGAAGCTGCTAGAACCGATTAAACCGGTTAACATCATGGCATGGGATATAAATGAAAAAGAAAAAAA
AACTCCGACGCCCCTTCCATCACATCATGTACTCTTCGCTGAACCGGG**CTCGAG**GGGGGGCCCGGTACCCGGGGATCCTCTAGAGTCGACCTGCAGGC
ATGCAAGCTTGGCGTAATCATGGTCATAGCTGTTTCCTGTGTGAAATTGTTATCCGCTCACAATTCCACACAACATACGAGCCGGAAGCATAAAGTGT
AAAGCCTGGGGTGCCTAATGAGTGAGCTAACTCACATTAATTGCGTTGCGCTCACTGCCCGCTTTCCAGTCGGGAAACCTGTCGTGCCAGCTGCATTA
ATGAATCGGCCAACGCGCGGGGAGAGGCGGTTTGCGTATTGGGCGCTCTTCCGCTTCCTCGCTCACTGACTCGCTGCGCTCGGTCGTTCGGCTGCGGC
GAGCGGTATCAGCTCACTCAAAGGCGGTAATACGGTTATCCACAGAATCAGGGGATAACGCAGGAAAGAACATGTGAGCAAAAGGCCAGCAAAAGGCC
AGGAACCGTAAAAAGGCCGCGTTGCTGGCGTTTTTCCATAGGCTCCGCCCCCCTGACGAGCATCACAAAAATCGACGCTCAAGTCAGAGGTGGCGAAA
CCCGACAGGACTATAAAGATACCAGGCGTTTCCCCCTGGAAGCTCCCTCGTGCGCTCTCCTGTTCCGACCCTGCCGCTTACCGGATACCTGTCCGCCT
TTCTCCCTTCGGGAAGCGTGGCGCTTTCTCATAGCTCACGCTGTAGGTATCTCAGTTCGGTGTAGGTCGTTCGCTCCAAGCTGGGCTGTGTGCACGAA
CCCCCCGTTCAGCCCGACCGCTGCGCCTTATCCGGTAACTATCGTCTTGAGTCCAACCCGGTAAGACACGACTTATCGCCACTGGCAGCAGCCACTGG
TAACAGGATTAGCAGAGCGAGGTATGTAGGCGGTGCTACAGAGTTCTTGAAGTGGTGGCCTAACTACGGCTACACTAGAAGAACAGTATTTGGTATCT
GCGCTCTGCTGAAGCCAGTTACCTTCGGAAAAAGAGTTGGTAGCTCTTGATCCGGCAAACAAACCACCGCTGGTAGCGGTGGTTTTTTTGTTTGCAAG
CAGCAGATTACGCGCAGAAAAAAAGGATCTCAAGAAGATCCTTTGATCTTTTCTACGGGGTCTGACGCTCAGTGGAACGAAAACTCACGTTAAGGGAT
TTTGGTCATGAGATTATCAAAAAGGATCTTCACCTAGATCCTTTTAAATTAAAAATGAAGTTTTAAATCAATCTAAAGTATATATGAGTAAACTTGGT
CTGACAGTTACCAATGCTTAATCAGTGAGGCACCTATCTCAGCGATCTGTCTATTTCGTTCATCCATAGTTGCCTGACTCCCCGTCGTGTAGATAACT
ACGATACGGGAGGGCTTACCATCTGGCCCCAGTGCTGCAATGATACCGCGAGACCCACGCTCACCGGCTCCAGATTTATCAGCAATAAACCAGCCAGC
CGGAAGGGCCGAGCGCAGAAGTGGTCCTGCAACTTTATCCGCCTCCATCCAGTCTATTAATTGTTGCCGGGAAGCTAGAGTAAGTAGTTCGCCAGTTA
ATAGTTTGCGCAACGTTGTTGCCATTGCTACAGGCATCGTGGTGTCACGCTCGTCGTTTGGTATGGCTTCATTCAGCTCCGGTTCCCAACGATCAAGG
CGAGTTACATGATCCCCCATGTTGTGCAAAAAAGCGGTTAGCTCCTTCGGTCCTCCGATCGTTGTCAGAAGTAAGTTGGCCGCAGTGTTATCACTCAT
GGTTATGGCAGCACTGCATAATTCTCTTACTGTCATGCCATCCGTAAGATGCTTTTCTGTGACTGGTGAGTACTCAACCAAGTCATTCTGAGAATAGT
GTATGCGGCGACCGAGTTGCTCTTGCCCGGCGTCAATACGGGATAATACCGCGCCACATAGCAGAACTTTAAAAGTGCTCATCATTGGAAAACGTTCT
TCGGGGCGAAAACTCTCAAGGATCTTACCGCTGTTGAGATCCAGTTCGATGTAACCCACTCGTGCACCCAACTGATCTTCAGCATCTTTTACTTTCAC
CAGCGTTTCTGGGTGAGCAAAAACAGGAAGGCAAAATGCCGCAAAAAAGGGAATAAGGGCGACACGGAAATGTTGAATACTCATACTCTTCCTTTTTC
AATATTATTGAAGCATTTATCAGGGTTATTGTCTCATGAGCGGATACATATTTGAATGTATTTAGAAAAATAAACAAATAGGGGTTCCGCGCACATTT
CCCCGAAAAGTGCCACCTGACGTCTAAGAAACCATTATTATCATGACATTAACCTATAAAAATAGGCGTATCACGAGGCCCTTTCGTC

pKH16 [pUC19–P_{HYP2}–P_{TEF}–*his5*–T_{TEF}–P_{GAL1}–tc3–HA–ORF_{HYP2}–T_{HYP2}]

TCGCGCGTTTCGGTGATGACGGTGAAAACCTCTGACACATGCAGCTCCCGGAGACGGTCACAGCTTGTCTGTAAGCGGATGCCGGGAGCAGACAAGCC
CGTCAGGGCGCGTCAGCGGGTGTTGGCGGGTGTCGGGGCTGGCTTAACTATGCGGCATCAGAGCAGATTGTACTGAGAGTGCACCATATGCGGTGTGA
AATACCGCACAGATGCGTAAGGAGAAAATACCGCATCAGGCGCCATTCGCCATTCAGGCTGCGCAACTGTTGGGAAGGGCGATCGGTGCGGGCCTCTT
CGCTATTACGCCAGCTGGCGAAAGGGGGATGTGCTGCAAGGCGATTAAGTTGGGTAACGCCAGGGTTTTCCCAGTCACGACGTTGTAAAACGACGGCC
AGTGAATTCCCTGACCATTGGCTTCGTTCTGGTGGCATTTTCTCTGAGGGGTGAGAACAGCAAAACGGGAACGGCGACTTGGAGTAGTATAACGCTGT
AGCTGGACGCTGAGGGGCCAAGGCCGGTAAAATTCTGCAACACCCAGACCTTGCTGCAGTGATATCATCCACCCATACCCCTCGATGTATTCCGTAGC
GTTATATCGGGTCCGTGTGAGGCGACAAGGGACCTCCCGCACCGCACATACCGCAGGAGCTAAAAGAAACGCCGTCGCCCGAAAAAAAGTTTCGAAGG
TGAAGGAACAGTGGTAATAAAAATTTTTTTCGTCGTCGTCGTCGATCGGCTTTTGGCCGCGTTGATCGTCGTGTTCCACGATTATATAATGCACAAGG
TTTTTGCCATATCTTGACATTGGAATCCCTCATTTCCTAAATTCCTCTCCTTCTATCAATTGCTTCCTTTCCTTCTTTCTAACGATTCTACTTCT**aga**
tctGTTTAGCTTGCCTCGTCCCCGCCGGGTCACCCGGCCAGCGACATGGAGGCCCAGAATACCCTCCTTGACAGTCTTGACGTGCGCAGCTCAGGGGC
ATGATGTGACTGTCGCCCGTACATTTAGCCCATACATCCCCATGTATAATCATTTGCATCCATACATTTTGATGGCCGCACGGCGCGAAGCAAAAATT
ACGGCTCCTCGCTGCAGACCTGCGAGCAGGGAAACGCTCCCCTCACAGACGCGTTGAATTGTCCCCACGCCGCGCCCCTGTAGAGAAATATAAAAGGT
TAGGATTTGCCACTGAGGTTCTTCTTTCATATACTTCCTTTTAAAATCTTGCTAGGATACAGTTCTCACATCACATCCGAACATAAACAACCATGGGT
AGGAGGGCTTTTGTAGAAAGAAATACGAACGAAACGAAAATCAGCGTTGCCATCGCTTTGGACAAAGCTCCCTTACCTGAAGAGTCGAATTTTATTGA
TGAACTTATAACTTCCAAGCATGCAAACCAAAAGGGAGAACAAGTAATCCAAGTAGACACGGGAATTGGATTCTTGGATCACATGTATCATGCACTGG
CTAAACATGCAGGCTGGAGCTTACGACTTTACTCAAGAGGTGATTTAATCATCGATGATCATCACACTGCAGAAGATACTGCTATTGCACTTGGTATT
GCATTCAAGCAGGCTATGGGTAACTTTGCCGGCGTTAAAAGATTTGGACATGCTTATTGTCCACTTGACGAAGCTCTTTCTAGAAGCGTAGTTGACTT
GTCGGGACGGCCCTATGCTGTTATCGATTTGGGATTAAAGCGTGAAAAGGTTGGGGAATTGTCCTGTGAAATGATCCCTCACTTACTATATTCCTTTT
CGGTAGCAGCTGGAATTACTTTGCATGTTACCTGCTTATATGGTAGTAATGACCATCATCGTGCTGAAAGCGCTTTTAAATCTCTGGCTGTTGCCATG
CGCGCGGCTACTAGTCTTACTGGAAGTTCTGAAGTCCCAAGCACGAAGGGAGTGTTGTAAAGAGTACTGACAATAAAAAGATTCTTGTTTTCAAGAAC
TTGTCATTTGTATAGTTTTTTTATATTGTAGTTGTTCTATTTTAATCAAATGTTAGCGTGATTTATATTTTTTTTCGCCTCGACATCATCTGCCCAGA
TGCGAAGTTAAGTGCGCAGAAAGTAATATCATGCGTCAATCGTATGTGAATGCTGGTCGCTATACTGCTGTCGATTCGATACTAACGCCGCCATCCAg
tttaaac**GAGCTC**TAGTACGGATTAGAAGCCGCCGAGCGGGTGACAGCCCTCCGAAGGAAGACTCTCCTCCGTGCGTCCTCGTCTTCACCGGTCGCGT
TCCTGAAACGCAGATGTGCCTCGCGCCGCACTGCTCCGAACAATAAAGATTCTACAATACTAGCTTTTATGGTTATGAAGAGGAAAAATTGGCAGTAA
CCTGGCCCCACAAACCTTCAAATGAACGAATCAAATTAACAACCATAGGATGATAATGCGATTAGTTTTTTAGCCTTATTTCTGGGGTAATTAATCAG
CGAAGCGATGATTTTTGATCTATTAACAGATATATAAATGCAAAAACTGCATAACCACTTTAACTAATACTTTCAACATTTTCGGTTTGTATTACTTC
TTATTCAAATGTAATAAAAGTATCAACAAAAAATTGTTAATATACCTCTATACTTTAACGTCAAGGAGAAAAAACCCCGCGGCCAATTATCTACTTAA
GGCCTAAAACATACCAGATCGCCACCCGCGCTTTAATCTGGAGAGGTGAAGAATACGACCACCTAGGCCTTAAGAACCGGTAAAACATACCAGATCGC
CACCCGCGCTTTAATCTGGAGAGGTGAAGAATACGACCACCTACCGGTCAACAACAACAACAACAACAACAACAACTCGAGGCCTAAAACATACCAGA
TCGCCACCCGCGCTTTAATCTGGAGAGGTGAAGAATACGACCACCTAGGCCTCGAGAACATATCTACCCATACGAGATCT**ATG**CACGGATCCTACCCA
TACGACGTCCCAGACTACGCTAAAGCTTCCGGGGGATGTCTGACGAAGAACATACCTTTGAAACTGCTGACGCTGGTTCCTCCGCCACCTACCCAAT
GCAATGTTCTGCCTTGAGAAAGAACGGTTTCGTTGTCATCAAGAGTAGACCATGTAAGATTGTCGACATGTCCACTTCTAAGACTGGTAAGCACGGTC
ACGCTAAAGTCCATTTGGTTGCCATTGATATCTTCACTGGTAAGAAGTTGGAAGATTTGTCTCCATCTACTCACAACATGGAAGTTCCAGTTGTCAAG
AGAAACGAATACCAATTGTTGGACATTGATGACGGTTTCTTGTCTTTGATGAACATGGACGGTGACACTAAGGATGATGTCAAGGCTCCAGAAGGTGA
ATTGGGTGACAGTTTGCAAACTGCTTTTGATGAAGGTAAGGACTTGATGGTTACCATCATCTCCGCTATGGGTGAAGAAGCCGCCATCTCCTTCAAGG
AAGCTGCTAGAACCGATTAAACCGGTTAACATCATGGCATGGGATATAAATGAAAAAAGAAAAAAAAACTCCGACGCCCCTTCCATCACATCATGTAC
TCTTCGCTGAACCGGG**CTCGAG**GGGGGGCCCGGTACCCGGGGATCCTCTAGAGTCGACCTGCAGGCATGCAAGCTTGGCGTAATCATGGTCATAGCTG
TTTCCTGTGTGAAATTGTTATCCGCTCACAATTCCACACAACATACGAGCCGGAAGCATAAAGTGTAAAGCCTGGGGTGCCTAATGAGTGAGCTAACT
CACATTAATTGCGTTGCGCTCACTGCCCGCTTTCCAGTCGGGAAACCTGTCGTGCCAGCTGCATTAATGAATCGGCCAACGCGCGGGGAGAGGCGGTT
TGCGTATTGGGCGCTCTTCCGCTTCCTCGCTCACTGACTCGCTGCGCTCGGTCGTTCGGCTGCGGCGAGCGGTATCAGCTCACTCAAAGGCGGTAATA
CGGTTATCCACAGAATCAGGGGATAACGCAGGAAAGAACATGTGAGCAAAAGGCCAGCAAAAGGCCAGGAACCGTAAAAAGGCCGCGTTGCTGGCGTT
TTTCCATAGGCTCCGCCCCCCTGACGAGCATCACAAAAATCGACGCTCAAGTCAGAGGTGGCGAAACCCGACAGGACTATAAAGATACCAGGCGTTTC
CCCCTGGAAGCTCCCTCGTGCGCTCTCCTGTTCCGACCCTGCCGCTTACCGGATACCTGTCCGCCTTTCTCCCTTCGGGAAGCGTGGCGCTTTCTCAT
AGCTCACGCTGTAGGTATCTCAGTTCGGTGTAGGTCGTTCGCTCCAAGCTGGGCTGTGTGCACGAACCCCCCGTTCAGCCCGACCGCTGCGCCTTATC
CGGTAACTATCGTCTTGAGTCCAACCCGGTAAGACACGACTTATCGCCACTGGCAGCAGCCACTGGTAACAGGATTAGCAGAGCGAGGTATGTAGGCG
GTGCTACAGAGTTCTTGAAGTGGTGGCCTAACTACGGCTACACTAGAAGAACAGTATTTGGTATCTGCGCTCTGCTGAAGCCAGTTACCTTCGGAAAA
AGAGTTGGTAGCTCTTGATCCGGCAAACAAACCACCGCTGGTAGCGGTGGTTTTTTTGTTTGCAAGCAGCAGATTACGCGCAGAAAAAAAGGATCTCA
AGAAGATCCTTTGATCTTTTCTACGGGGTCTGACGCTCAGTGGAACGAAAACTCACGTTAAGGGATTTTGGTCATGAGATTATCAAAAAGGATCTTCA
CCTAGATCCTTTTAAATTAAAAATGAAGTTTTAAATCAATCTAAAGTATATATGAGTAAACTTGGTCTGACAGTTACCAATGCTTAATCAGTGAGGCA
CCTATCTCAGCGATCTGTCTATTTCGTTCATCCATAGTTGCCTGACTCCCCGTCGTGTAGATAACTACGATACGGGAGGGCTTACCATCTGGCCCCAG
TGCTGCAATGATACCGCGAGACCCACGCTCACCGGCTCCAGATTTATCAGCAATAAACCAGCCAGCCGGAAGGGCCGAGCGCAGAAGTGGTCCTGCAA
CTTTATCCGCCTCCATCCAGTCTATTAATTGTTGCCGGGAAGCTAGAGTAAGTAGTTCGCCAGTTAATAGTTTGCGCAACGTTGTTGCCATTGCTACA
GGCATCGTGGTGTCACGCTCGTCGTTTGGTATGGCTTCATTCAGCTCCGGTTCCCAACGATCAAGGCGAGTTACATGATCCCCCATGTTGTGCAAAAA
AGCGGTTAGCTCCTTCGGTCCTCCGATCGTTGTCAGAAGTAAGTTGGCCGCAGTGTTATCACTCATGGTTATGGCAGCACTGCATAATTCTCTTACTG
TCATGCCATCCGTAAGATGCTTTTCTGTGACTGGTGAGTACTCAACCAAGTCATTCTGAGAATAGTGTATGCGGCGACCGAGTTGCTCTTGCCCGGCG
TCAATACGGGATAATACCGCGCCACATAGCAGAACTTTAAAAGTGCTCATCATTGGAAAACGTTCTTCGGGGCGAAAACTCTCAAGGATCTTACCGCT
GTTGAGATCCAGTTCGATGTAACCCACTCGTGCACCCAACTGATCTTCAGCATCTTTTACTTTCACCAGCGTTTCTGGGTGAGCAAAAACAGGAAGGC
AAAATGCCGCAAAAAAGGGAATAAGGGCGACACGGAAATGTTGAATACTCATACTCTTCCTTTTTCAATATTATTGAAGCATTTATCAGGGTTATTGT
CTCATGAGCGGATACATATTTGAATGTATTTAGAAAAATAAACAAATAGGGGTTCCGCGCACATTTCCCCGAAAAGTGCCACCTGACGTCTAAGAAAC
CATTATTATCATGACATTAACCTATAAAAATAGGCGTATCACGAGGCCCTTTCGTC

Table S1: Quantification of anti-Myc western blot signals of blot shown in Fig. 4

Strain	Band(s)	Signal	%	% RFS	Mean	SD	T-test vs. WT (p)
**Wild type 1**	Full-length	1391147	14,47	14,47			
	truncated	8222782	85,53				
	Total	9613929	100,00				
**Wild type 2**	truncated	1395516	14,01	14,01	14.48	0,47	
	Full-length	8565407	85,99				
	Total	9960923	100,00				
**Wild type 3**	truncated	1518780	14,95	14,95			
	Full-length	8642364	85.05				
	Total	10161144	100,00				
***DYS1* -SD 1**	Full-length	434763	4,91	4,91			
	truncated	8416616	95,09				
	Total	8851379	100,00				
***DYS1* -SD 2**	truncated	508256	5,42	5,42	5,27	0,32	0,0000296
	Full-length	8860794	96,58				
	Total	9369050	100,00				
***DYS1* -SD 3**	truncated	478043	5,49	5,49			
	Full-length	8222782	94,51				
	Total	8700825	100,00				
***HYP2* -SD 1**	Full-length	256957	4,42	4,42			
	truncated	5551927	95,58				
	Total	5808884	100,00				
***HYP2* -SD 2**	truncated	433732	5,46	5,46	5,08	0,57	0,0000337
	Full-length	7504955	94,54				
	Total	7938687	100,00				
***HYP2* -SD 3**	truncated	430305	5,35	5,35			
	Full-length	7611366	94.65				
	Total	8041671	100,00				

Table S2 – *S. cerevisiae* strains used in this study

Strain*	Relevant Genotype	Source / Reference
WCG4a or -α	*his3-11,15, leu2-3,112, ura3-52*	[63]
KH27-1B,-5C,-9C**	*hyp1Δ::KanMX6, pKH16::His3MX6 (in HYP2), pFO1::URA3, LEU2, his3-11,15*	This work
KH29-3A	*hyp1Δ::KanMX6, pKH16::His3MX6 (in HYP2), pFO2::URA3, LEU2, his3-11,15*	This work
KH29-2B	*hyp1Δ::KanMX6, pKH16::His3MX6 (in HYP2), LEU2, his3-11, ura3-52*	This work
KH32-10D,-11D,-11B**	*hyp1Δ::KanMX6, pKH13::His3MX6 (in DYS1), pFO2::URA3, LEU2, his3-11,15*	This work
KH33-4C,-5D,-9B**	*hyp1Δ::KanMX6, pKH13::His3MX6 (in DYS1), pFO1::URA3, LEU2, his3-11,15*	This work
KH30	*hyp1Δ::KanMX6, pFO2::URA3, LEU2, HIS3*	This work
KH31	*hyp1Δ::KanMX6, pFO1::URA3, LEU2, HIS3*	This work
JD411-1A,4B**	*pFO1::URA3, LEU2, HIS3*	This work
JD403-12C	*pFO2::URA3, LEU2, HIS3*	This work

**All strains are congenic derivatives of WCG4a or WCG4-α*
***Congenic spore clones obtained by tetrad dissection*

Table S3 –Plasmids used in this study

Name	Relevant characteristics	Derivative of / reference
pKH11	P_{HYP2}-*HYP2*, *CEN/LEU2*	pRS315
pKH12	P_{HYP2}-*HA-HYP2*, *CEN/LEU2*	pRS315
pKH18	P_{HYP2}-*hyp2*^{K51R}, *CEN/LEU2*	pRS315
pKH13	5´Δ-P_{DYS1}-*His3MX6*-$P_{GAL1\text{-}tc3}$-Ub-R-HA-*DYS1*-3´Δ	pJET1.2
pKH14	5´Δ-P_{DYS1}- *His3MX6*-$P_{GAL1\text{-}tc3}$-HA-*DYS1*-3´Δ	pJET1.2
pKH15	5´Δ-P_{HYP2}- *His3MX6*-$P_{GAL1\text{-}tc3}$-Ub-R-HA-*HYP2*-T_{HYP2}	pUC19
pKH16	5´Δ-P_{HYP2}- *His3MX6*-$P_{GAL1\text{-}tc3}$-HA-*HYP2*-T_{HYP2}	pUC21
pFO1	P_{ADH1}-*2xMYC-DHFR-RFS-2xHA*-T_{CYC1}*::URA3*	pLD57
pFO2	P_{ADH1}-*2xMYC-DHFR-IF-2xHA*::T_{CYC1}*::URA3*	pLD57
pLD57	P_{URA3}-*URA3*-$T_{URA3\ (with\ polylinker)}$	pUC21
pRS315	*CEN/LEU2*	[64]
pUC19	*E. coli* cloning vector with Amp^R	[66]
pUC21	*E. coli* cloning vector with Amp^R	[65]
pJET1.2	*E. coli* cloning vector with Amp^R	Thermofisher
pFA6-His3MX6	*His3MX6*	[56] Longtine 1998
pFA6-KanMX6	*KanMX6*	[56] Longtine 1998
pPW66R	*Ub-R-DHFR*^{td}*-CDC28*	[51] Dohmen 1994

Table S4 – Antibodies used in this study

Antibody	Host	Dilution	Source
Anti-HA (3F10)	rat	1:1000	Merck
Anti-Myc (9B11)	mouse	1:1000	Cell Signaling Technology
Anti-Hypusine	rabbit	1:2000	Merck
Anti-Rabbit, Alexa Fluor Plus 800 (A32735)	goat	1:5000	Thermo Fisher
Anti-Mouse, Alexa Fluor Plus 680 (A32729)	goat	1:20000	Thermo Fisher
Anti-Mouse, Alexa Fluor 680 (A21096)	goat	1:5000	Thermo Fisher
